# Pre-pregnancy and gestational cardiometabolic disorders and risk of preterm birth and infant mortality

**DOI:** 10.7189/jogh.15.04333

**Published:** 2025-12-05

**Authors:** Yangyang Cheng, Mika Kivimäki, Yue Zhang, Rodrigo M Carrillo-Larco, Xiaochen Dai, Yaogang Wang, Xiaolin Xu

**Affiliations:** 1 School of Public Health, The Second Affiliated Hospital, Zhejiang University School of Medicine, Hangzhou, Zhejiang, China; 2 UCL Brain Sciences, University College London, London, UK; 3 Emory Global Diabetes Research Center, Emory University, Atlanta, Georgia, USA; 4 Hubert Department of Global Health, Rollins School of Public Health, Emory University, Atlanta, Georgia, USA; 5 Institute for Health Metrics and Evaluation, University of Washington, Seattle, Washington, USA; 6 Department of Health Metrics Sciences, School of Medicine, University of Washington, Seattle, Washington, USA; 7 School of Public Health, Tianjin Medical University, Tianjin, China; 8 School of Integrative Medicine, Public Health Science and Engineering College, Tianjin University of Traditional Chinese Medicine, Tianjin, China; 9 National Institute of Health Data Science at Peking University, Peking University, Beijing, China; 10 School of Public Health, Faculty of Medicine, The University of Queensland, Brisbane, Queensland, Australia

## Abstract

**Background:**

Cardiometabolic disorders (CMDs) are common in pregnancy and can harm the offspring’s health. While prior studies have explored clustered cardiometabolic risks in pregnancy, most have focused on a limited number of conditions or a single period. We aimed to examine the associations of individual, multiple, and separate and combined patterns of six pre-pregnancy and gestational CMDs with preterm birth and infant mortality.

**Methods:**

Using data from US National Vital Statistics System (2014–2020), we analysed pre-pregnancy CMDs (body mass index, diabetes, hypertension) and gestational CMDs (gestational weight gain, gestational diabetes, hypertensive disorders). We estimated the prevalence and time trends in CMDs using Joinpoint regression models and examined associations with preterm birth and infant mortality using multivariable logistic regression.

**Results:**

Among 24 447 869 mother-infant pairs, 1 932 716 (7.9%) were preterm births and 108 891 (0.5%) were infant deaths. Prevalence rates of most multi-CMD patterns increased significantly. There was a dose-response association between the number of pre-pregnancy and gestational CMDs and the risk of preterm birth and infant mortality (*P* for trend <0.001). Co-occurring pre-pregnancy diabetes and hypertension showed the strongest associations with preterm birth (odds ratio (OR) = 10.52; 95% CI = 9.71–11.40) and infant mortality (OR = 3.93; 95% CI = 2.99–5.18). Co-occurring inadequate gestational weight gain, gestational diabetes and hypertensive disorders showed the strongest association with preterm birth (OR = 4.57; 95% CI = 4.46–4.68). Specific combinations of pre-pregnancy and gestational CMD patterns such as pre-pregnancy diabetes and developed additional gestational hypertensive disorders experienced highest risk of preterm birth (OR = 18.80; 95% CI = 17.38–20.35).

**Conclusions:**

Increasing prevalence of multiple CMDs was associated with higher risks of preterm birth and infant mortality, emphasising the need for enhanced prevention and management of cardiometabolic health before and during pregnancy.

Delayed childbearing has risen in recent years, increasing the prevalence and coexistence of cardiometabolic disorders (CMDs) in women’s peripartum period [1–3]. Obesity, diabetes, and hypertensive disorders, the most common CMDs in pregnancy [4,5], are associated with adverse birth outcomes [4]. Among these outcomes, preterm birth and infant mortality are crucial indicators of reproductive health worldwide [6,7], and their prevention is of paramount important [8].

Previous studies have investigated the associations of CMDs such as diabetes [9,10], hypertension [11,12] present before or during pregnancy, pre-pregnancy body mass index (BMI) [13,14], and gestational weight gain (GWG) [15,16] with preterm birth and neonatal mortality. However, important gaps remain. First, most research on CMDs focused on preterm birth, yielding conflicting results [16,17], while evidence linking CMDs and infant mortality has rarely been reported.

Second, previous studies on CMDs in pregnancy were limited to single conditions examined in isolation. As CMDs become more prevalent and often co-exist among women of higher childbearing ages [18] the accumulation of multiple CMDs might increase the risks of adverse pregnancy outcomes. In particular, multiple CMDs are likely to have synergistic effects through shared mechanisms, such as insulin resistance, chronic inflammation, placental dysfunction, oxidative stress and endothelial dysfunction [19]. A previous study found that there were graded associations between greater pre-pregnancy cardiovascular risk factors (non-ideal BMI, diabetes, and hypertension) and adverse maternal and offspring outcomes [20]. However, no such evidence is available for gestational cardiometabolic risk factors.

Third, CMDs before and during pregnancy may be interrelated [21], but previous studies were limited to measurements of specific pairs of co-occurring CMDs or a single period, such as only pre-pregnancy CMDs (chronic hypertension and pregestational diabetes) [22,23], only gestational CMDs (GDM and hypertensive disorders during pregnancy, HDP) [24], or only the combination of single CMDs (pre-pregnancy BMI and GWG) [25], with few studies examining the joint associations of multiple pre-pregnancy and gestational CMDs.

To address these gaps, we aimed to examine the associations of individual, multiple, separate and combined patterns of pre-pregnancy and gestational CMDs with preterm birth and infant mortality in a large-scale, nationally representative USA population, 2014–2020.

## METHODS

### Study design and participants

This study used records from the US National Vital Statistics System (NVSS), collected by the National Center for Health Statistics. The NVSS provides individual-level records on all live births and infant deaths across the 50 states and the District of Columbia, collected through standardised maternal and facility worksheets.

**Figure 1:**
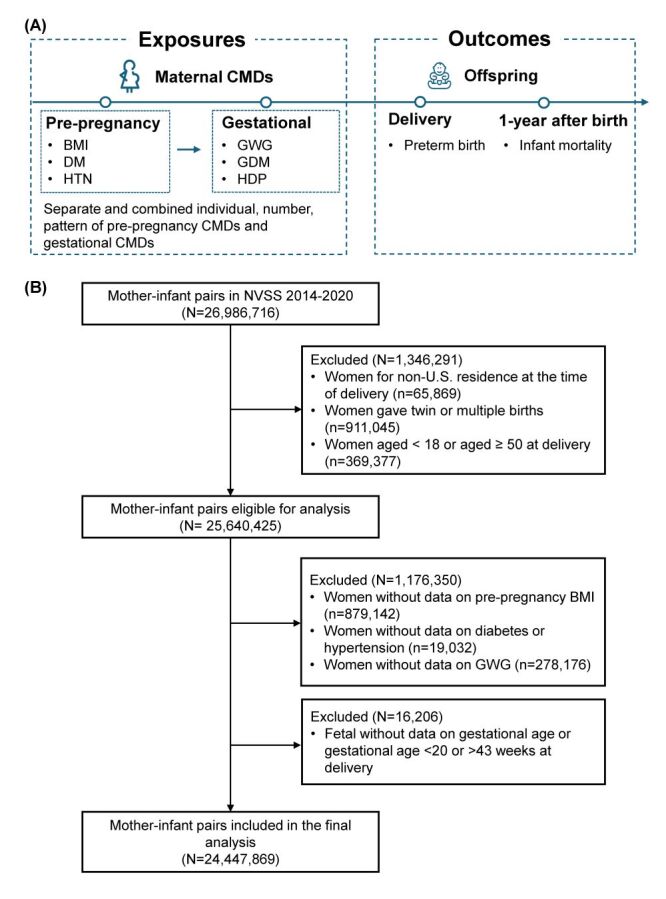
Flowchart of the study design. **Panel A.** The study design and concept map. **Panel B.** The participants selection process. BMI – body mass index, CMD – cardiometabolic disorder, GDM – gestational diabetes mellitus, GWG – gestational weight gain, HDP – hypertensive disorders during pregnancy, NVSS – National Vital Statistics System.

We obtained records of NVSS birth cohort linked birth and infant death databases between 2014 and 2020. All mothers who had a singleton birth, aged 18–49 years, and were USA residence at delivery were included. Records with incomplete exposure or outcome data were excluded. Details of participant inclusion are shown in **Figure 1**. This study followed the Strengthening the Reporting of Observational Studies in Epidemiology (STROBE) guideline (Checklist S1 in the **Online Supplementary Document**).

### Assessment of pre-pregnancy and gestational cardiometabolic disorders

Exposures included three pre-pregnancy CMDs (BMI, diabetes, and hypertension) and three gestational CMDs (GWG, GDM, and HDP). Information on mother’s height and pre-pregnancy weight was collected from the mother’s worksheet; information on mother’s weight at delivery, diabetes (pre-pregnancy or gestational) and hypertension (pre-pregnancy or gestational) were collected directly from the medical record using the facility worksheet.

We calculated BMI as pre-pregnancy weight (kg) divided by the square of height (meters) and classified into underweight (<18.5 kg/m^2^), normal (18.5–24.9 kg/m^2^), overweight (25.0–29.9 kg/m^2^), or obese (≥30.0 kg/m^2^) [20]. Normal weight was defined as ideal, and the others as non-ideal BMI. Pre-pregnancy diabetes was defined as having type 1 or type 2 diabetes diagnosed prior to pregnancy. Pre-pregnancy hypertension was defined as high blood pressure prior to the pregnancy [26].

We calculated GWG as the difference between delivery and pre-pregnancy weight. Following the 2009 USA Institute of Medicine guidelines, which recommend BMI-specific ranges (underweight: 28–40 pounds (lbs); normal weight: 25–35 lbs; overweight: 15–25 lbs; obese: 11–20 lbs). We classified GWG as inadequate (below recommendations), adequate (within recommendations), or excessive (above recommendations) [27]. Adequate GWG was defined as ideal, and inadequate or excessive GWG as non-ideal. Newly diagnosed diabetes during pregnancy was defined as GDM. For HDP, the condition was defined as newly diagnosed hypertension during pregnancy, including gestational hypertension and preeclampsia, no matter with or without eclampsia [28].

We initially constructed two sets of CMD pattern variables: a 16-category for pre-pregnancy and a 12-category for gestational. For descriptive analysis, each set was subsequently collapsed into eight categories. We then generated a 108-category variable including all combination patterns of pre-pregnancy and gestational CMDs. To optimise model fitting, we merged certain categories (underweight, overweight, and obese as non-ideal BMI), which ultimately resulted in a refined 54-category variable for analyses.

### Assessment of offspring outcomes

Information on gestational age was based on the obstetric estimate of gestation at delivery, collected directly from the medical record. We defined preterm birth as live birth occurring before 37 weeks of gestation. Information on infant mortality under one year of age was collected from death certificate.

### Assessment of covariates

We considered the following covariates. maternal age at delivery (<20, 20–24, 25–29, 30–34, 35–39, ≥40), race/ethnicity (non-Hispanic White, non-Hispanic Black, Hispanic, other), marital status between conception and delivery (married, unmarried), educational level (below high school, high school, above high school), parity (nulliparous, multiparous), the time of prenatal care initiation (1st to 3rd month, 4th to 6th month, 7th to final month, no prenatal care), the total number of prenatal care visits (0, 1–4, 5–9, ≥10 times), smoking status before or during pregnancy (yes or no), infant sex (male or female). Covariates with missing values were classified into a separate category of unknown.

### Statistical analysis

The characteristics of the study population were presented as number and percentages by preterm birth and infant mortality, and differences among groups were tested by analysis of χ^2^ test. The age-adjusted prevalence rates per 100 mothers of individual, multiple, and patterns of pre-pregnancy CMDs and gestational CMDs were calculated and the average annual percentage change (AAPC) with 95% confidence interval (CI) were compared over time from 2014 to 2020 to describe relative changes in prevalence rates. The transmission patterns of CMDs from pre-pregnancy to gestation were presented in a Sankey diagram. We also conducted separate logistic models to assess the associations between pre-pregnancy and gestational CMDs.

Multivariable-adjusted logistic regression models were used to estimate the odds ratios (ORs) and 95% CI for each outcome, adjusting for maternal age, race/ethnicity, marital status, educational level, parity, time of prenatal care initiation, total number of prenatal care visits, smoking before or during pregnancy, infant sex. Our modelling included four steps. First, we examined the associations of each single pre-pregnancy and gestational CMDs with preterm birth and infant mortality individually. Second, we assessed the associations of the number of pre-pregnancy and gestational CMDs with preterm birth and infant mortality. Moreover, we added CMDs number as a continuous variable in the model to examine its potential dose-response effect on the outcomes. Third, we examined the associations of patterns of pre-pregnancy CMDs and gestational CMDs separately with the two outcomes. Fourth, we evaluated the associations of the 54 combinations of pre-pregnancy and gestational CMDs with the outcomes. Additionally, to address potential model instability due to sparse data in some exposure combinations, we performed a sensitivity analysis using Firth’s logistic regression on a 10% random subsample.

A series of additional analyses were conducted to examine the robustness of the main results. First, we classified preterm birth as moderately (32–36 weeks), very (28–31 weeks), or extremely (20–27 weeks) preterm birth, and infant mortality as neonatal (deaths within 0–27 days) or post-neonatal mortality (deaths within 28–364 days), to evaluate the association of pre-pregnancy CMDs and gestational CMDs with the subclassification of preterm birth and infant mortality. Second, we conducted sensitivity analyses by excluding women with missing covariates and excluding multiparous women. Third, we did subgroup analyses according to maternal age (<35 and ≥35 years), race/ethnicity (non-Hispanic White and the others), marital status (married and unmarried), and educational level (under college and college or above).

We used Joinpoint Regression Program, version 5.0.2 (National Cancer Institute, Bethesda, Maryland, USA), SAS, version 9.4 (SAS Institute Inc., North Carolina, USA), and *R*, version 4.3.0 (R Core Team, Vienna, Austria) for all statistical analyses. Two-sided *P* < 0.05 were considered statistically significant.

## RESULTS

### Population characteristics

A total of 24 447 869 mother-infant pairs were included in this study (**Table 1**). Of these, 1 932 716 (7.9%) were preterm births and 108 891 (0.5%) infant deaths during 2014–2020. Mothers with preterm births or infant deaths were more likely to be non-Hispanic Black, unmarried, lower educated, have fewer prenatal care visits, and to be smokers before or during pregnancy. Characteristics of excluded participants were more likely to be older (Table S1 in the **Online Supplementary Document**).

**Table 1:** Characteristics of the study population (n = 24 447 869)

Characteristics	Total	Preterm birth*		*P-*value	Infant mortality*		*P-*value
		**Yes**	**No**		**Yes**	**No**	
Overall, n (%)	24 447 869 (100.0)	1 932 716 (7.9)	22 515 153 (92.1)		108 891 (0.5)	24 338 978 (99.5)	
Maternal age (years), n (%)				<0.001			<0.001
*<20*	972 867 (4.0)	85 103 (4.4)	887 764 (3.9)		6647 (6.1)	966 220 (4.0)	
*20–24*	5 032 357 (20.6)	400 239 (20.7)	4 632 118 (20.6)		28 033 (25.7)	5 004 324 (20.6)	
*25–29*	7 193 869 (29.4)	530 701 (27.5)	6 663 168 (29.6)		30 843 (28.3)	7 163 026 (29.4)	
*30–34*	6 989 754 (28.6)	520 198 (26.9)	6 469 556 (28.7)		24 935 (22.9)	6 964 819 (28.6)	
*35–39*	3 485 446 (14.3)	308 663 (16.0)	3 176 783 (14.1)		13 911 (12.8)	3 471 535 (14.3)	
40–49	773 576 (3.2)	87 812 (4.5)	685 764 (3.1)		4522 (4.2)	769 054 (3.2)	
Race/ethnicity, n (%)				<0.001			<0.001
*Hispanic*	5 702 836 (23.3)	458 506 (23.7)	5 244 330 (23.3)		22 532 (20.7)	5 680 304 (23.3)	
*Non-Hispanic White*	12 812 556 (52.4)	893 543 (46.2)	11 919 013 (52.9)		47 849 (43.9)	12 764 707 (52.5)	
*Non-Hispanic Black*	3 413 016 (14.0)	390 040 (20.2)	3 022 976 (13.4)		28 456 (26.1)	3 384 560 (13.9)	
*Other*	2 324 673 (9.5)	175 023 (9.1)	2 149 650 (9.6)		8718 (8.0)	2 315 955 (9.5)	
*Unknown*	194 788 (0.8)	15 604 (0.8)	179 184 (0.8)		1336 (1.2)	193 452 (0.8)	
Marital status, n (%)				<0.001			<0.001
*Married*	13 810 417 (56.5)	958 427 (49.6)	12 851 990 (57.1)		46 296 (42.5)	13 764 121 (56.6)	
*Unmarried*	8 967 019 (36.7)	855 350 (44.3)	8 111 669 (36.0)		57 095 (52.4)	8 909 924 (36.6)	
*Unknown*	1 670 433 (6.8)	118 939 (6.2)	1 551 494 (6.9)		5500 (5.1)	1 664 933 (6.8)	
Educational level, n (%)				<0.001			<0.001
*Under high school*	2 966 409 (12.1)	281 267 (14.6)	2 685 142 (11.9)		18 687 (17.2)	2 947 722 (12.1)	
*High school*	11 278 751 (46.1)	979 034 (50.7)	10 299 717 (45.8)		60 056 (55.2)	11 218 695 (46.1)	
*Above high school*	9 925 964 (40.6)	647 787 (33.5)	9 278 177 (41.2)		27 265 (25.0)	9 898 699 (40.7)	
*Unknown*	276 745 (1.1)	24 628 (1.3)	252 117 (1.1)		2883 (2.7)	273 862 (1.1)	
Parity, n (%)				<0.001			<0.001
*Nulliparous*	9 313 522 (38.1)	742 018 (38.4)	8 571 504 (38.1)		41 120 (37.8)	9 272 402 (38.1)	
*Multiparous*	15 079 343 (61.7)	1 185 432 (61.3)	13 893 911 (61.7)		67 347 (61.9)	15 011 996 (61.7)	
*Unknown*	55 004 (0.2)	5266 (0.3)	49 738 (0.2)		424 (0.4)	54 580 (0.2)	
Time of initiation of prenatal care, n (%)				<0.001			<0.001
*1st to 3rd month*	18 647 180 (76.3)	1 427 378 (73.9)	17 219 802 (76.5)		71 148 (65.3)	18 576 032 (76.3)	
*4th to 6th month*	3 921 111 (16.0)	294 728 (15.3)	3 626 383 (16.1)		21 062 (19.3)	3 900 049 (16.0)	
*7th to final month*	1 035 912 (4.2)	65 381 (3.4)	970 531 (4.3)		4905 (4.5)	1 031 007 (4.2)	
*No prenatal care*	342 254 (1.4)	76 386 (4.0)	265 868 (1.2)		6263 (5.8)	335 991 (1.4)	
*Unknown*	501 412 (2.1)	68 843 (3.6)	432 569 (1.9)		5513 (5.1)	495 899 (2.0)	
Total number of prenatal care visits, n (%)				<0.001			<0.001
*0 visit*	342 253 (1.4)	76 386 (4.0)	265 867 (1.2)		6263 (5.8)	335 990 (1.4)	
*1–4 visits*	850 074 (3.5)	168 343 (8.7)	681 731 (3.0)		20 251 (18.6)	829 823 (3.4)	
*5–9 visits*	5 030 394 (20.6)	713 715 (36.9)	4 316 679 (19.2)		36 305 (33.3)	4 994 089 (20.5)	
*≥10 visits*	17 724 452 (72.5)	906 882 (46.9)	16 817 570 (74.7)		40 865 (37.5)	17 683 587 (72.7)	
*Unknown*	500 696 (2.1)	67 390 (3.5)	433 306 (1.9)		5207 (4.8)	495 489 (2.0)	
Smoking before pregnancy, n (%)				<0.001			<0.001
*No*	22 125 187 (90.5)	1 686 742 (87.3)	20 438 445 (90.8)		90 186 (82.8)	22 035 001 (90.5)	
*Yes*	2 185 714 (8.9)	232 642 (12.0)	1 953 072 (8.7)		17 590 (16.2)	2 168 124 (8.9)	
*Unknown*	136 968 (0.6)	13 332 (0.7)	123 636 (0.6)		1115 (1.0)	135 853 (0.6)	
Smoking during pregnancy, n (%)				<0.001			<0.001
*No*	22 634 813 (92.6)	1 726 782 (89.3)	20 908 031 (92.9)		92 764 (85.2)	22 542 049 (92.6)	
*Yes*	1 678 364 (6.9)	192 763 (10.0)	1 485 601 (6.6)		15 017 (13.8)	1 663 347 (6.8)	
*Unknown*	134 692 (0.6)	13 171 (0.7)	121 521 (0.5)		1110 (1.0)	133 582 (0.6)	
Infant sex, n (%)				<0.001			<0.001
*Male*	12 514 112 (51.2)	1 041 733 (53.9)	11 472 379 (51.0)		60 280 (55.4)	12 453 832 (51.2)	
*Female*	11 993 757 (48.8)	890 983 (46.1)	11 042 774 (49.1)		48 611 (44.6)	11 885 146 (48.8)	

*χ^2^ test were used to compare differences across groups for preterm birth and infant mortality, respectively.

### Prevalence and trends of pre-pregnancy and gestational CMDs

Among the women, 57.2% had one or more pre-pregnancy CMDs. The most prevalent pre-pregnancy CMD pattern was only non-ideal BMI (54.6%; overweight = 25.8%; obese = 25.5%; underweight = 3.3%), followed by obese combined with hypertension (1.1%), and obese combined with diabetes (0.4%) (**Table 2**). Regarding gestational CMDs, 71.8% of the women had one or more conditions, including those with non-ideal GWG (59.7%; excessive GWG = 40.6%; inadequate GWG = 19.1%), excessive GWG combined HDP (3.4%), excessive GWG combined GDM (2.3%) and others (**Table 3**). Among the women, 84.8% had at least one pre-pregnancy or gestational CMDs and 46.8% had two or more CMDs.

**Table 2:** Associations of pre-pregnancy cardiometabolic disorders with preterm birth and infant mortality (n = 24 447 869)

Pre-pregnancy CMDs	Total, n (%)	Preterm birth		Infant mortality	
		**Cases, n (%)**	OR (95% CI)*	**Cases, n (%)**	OR (95% CI)*
**Individual CMDs†**					
BMI					
*Underweight*	807 332 (3.3)	77 409 (9.6)	1.31 (1.30–1.32)	3935 (0.5)	1.07 (1.03–1.10)
*Normal weight*	10 560 623 (43.2)	743 248 (7.0)	1.00 (Reference)	39 592 (0.4)	1.00 (Reference)
*Overweight*	6 463 431 (26.4)	490 713 (7.6)	1.06 (1.06–1.07)	27 650 (0.4)	1.13 (1.11–1.15)
*Obese*	6 616 483 (27.1)	621 346 (9.4)	1.33 (1.32–1.33)	37 714 (0.6)	1.44 (1.42–1.46)
DM					
*No*	24 226 917 (99.1)	1 876 835 (7.8)	1.00 (Reference)	106 536 (0.4)	1.00 (Reference)
*Yes*	220 952 (0.9)	55 881 (25.3)	4.14 (4.10–4.18)	4283 (0.9)	2.32 (2.23–2.42)
HTN					
*No*	23 980 072 (98.1)	1 834 443 (7.7)	1.00 (Reference)	104 608 (0.4)	1.00 (Reference)
*Yes*	467 797 (1.9)	98 273 (21.0)	3.03 (3.01–3.05)	1544 (1.0)	1.80 (1.74–1.86)
**Number of CMDs**					
0	10 448 577 (42.7)	718 432 (6.9)	1.00 (Reference)	38 642 (0.4)	1.00 (Reference)
1	13 455 793 (55.0)	1 096 690 (8.2)	1.17 (1.17–1.18)	65 073 (0.5)	1.25 (1.24–1.27)
2	510 295 (2.1)	105 850 (20.7)	3.43 (3.40–3.45)	4664 (0.9)	2.20 (2.13–2.27)
3	33 204 (0.1)	11 744 (35.4)	6.99 (6.82–7.16)	512 (1.5)	3.42 (3.13–3.74)
Continuous‡			1.41 (1.40–1.41)		1.34 (1.33–1.36)
P for trend			<0.001		<0.001
**Patterns of CMDs**					
No CMDs	10 448 577 (42.7)	718 432 (6.9)	1.00 (Reference)	38 642 (0.4)	1.00 (Reference)
Only underweight	801 870 (3.3)	75 947 (9.5)	1.32 (1.31–1.33)	3864 (0.5)	1.07 (1.03–1.10)
Only overweight	6 318 079 (25.8)	459 312 (7.3)	1.04 (1.04–1.05)	26 369 (0.4)	1.12 (1.10–1.14)
Only obese	6 226 503 (25.5)	537 824 (8.6)	1.25 (1.24–1.25)	33 944 (0.6)	1.41 (1.39–1.43)
Only DM	38 235 (0.2)	9127 (23.9)	4.60 (4.48–4.71)	331 (0.9)	2.30 (2.06–2.56)
Only HTN	71 106 (0.3)	14 480 (20.4)	3.18 (3.12–3.24)	565 (0.8)	1.71 (1.57–1.86)
Underweight + DM	1918 (0.0)	513 (26.8)	4.84 (4.35–5.39)	23 (1.2)	2.51 (1.65–3.81)
Overweight + DM	45 715 (0.2)	10 600 (23.2)	4.32 (4.23–4.42)	385 (0.8)	2.29 (2.07–2.54)
Obese + DM	99 175 (0.4)	22 688 (22.9)	4.10 (4.03–4.16)	1050 (1.1)	2.77 (2.61–2.95)
Underweight + HTN	3436 (0.0)	901 (26.2)	4.07 (3.75–4.41)	47 (1.4)	2.36 (1.76–3.16)
Overweight + HTN	94 301 (0.4)	18 682 (19.8)	3.10 (3.05–3.16)	807 (0.9)	1.96 (1.83–2.11)
Obese + HTN	263 045 (1.1)	51 257 (19.5)	3.12 (3.08–3.15)	2298 (0.9)	2.07 (1.98–2.16)
DM + HTN	2705 (0.0)	1209 (44.7)	10.52 (9.71–11.40)	54 (2.0)	3.93 (2.99–5.18)
Underweight + DM + HTN	108 (0.0)	48 (44.4)	9.82 (6.56–14.71)	1 (0.9)	1.50 (0.21–10.96)
Overweight + DM + HTN	5336 (0.0)	2119 (39.7)	8.36 (7.89–8.86)	89 (1.7)	3.45 (2.79–4.27)
Obese + DM + HTN	27 760 (0.1)	9577 (34.5)	6.76 (6.58–6.94)	422 (1.5)	3.45 (3.13–3.81)

BMI – body mass index, CI – confidence interval, CMD – cardiometabolic disorder, DM – diabetes mellitus, HTN – hypertension, OR – odds ratio

*Models were adjusted for maternal age, race/ethnicity, marital status, educational level, parity, time of initiation of prenatal care, total number of prenatal care visits, smoking before or during pregnancy, infant sex.

†Three separate models were built for each individual CMDs.

‡Models were constructed by considering the total CMD number as a continuous variable.

**Table 3:** Associations of gestational cardiometabolic disorders with preterm birth and infant mortality (n = 24 447 869)

Gestational CMDs	Total, n (%)	Preterm birth		Infant mortality	
		**Cases, n (%)**	OR (95% CI)*	**Cases, n (%)**	OR (95% CI)*
**Individual CMDs†**					
GWG					
*Inadequate*	5 320 898 (21.8)	644 596 (12.1)	1.53 (1.53–1.54)	46 259 (0.9)	1.91 (1.89–1.94)
*Adequate*	7 709 265 (31.5)	585 363 (7.6)	1.00 (Reference)	28 722 (0.4)	1.00 (Reference)
*Excessive*	11 417 706 (46.7)	702 757 (6.2)	0.81 (0.80–0.81)	33 910 (0.3)	0.81 (0.79–0.82)
GDM					
*No*	22 881 018 (93.6)	1 756 742 (7.7)	1.00 (Reference)	103 609 (0.5)	1.00 (Reference)
*Yes*	1 566 851 (6.4)	175 974 (11.2)	1.65 (1.64–1.66)	5282 (0.3)	0.88 (0.85–0.90)
HDP					
*No*	22 875 996 (93.6)	1 630 671 (7.1)	1.00 (Reference)	100 618 (0.4)	1.00 (Reference)
*Yes*	1 571 873 (6.4)	302 045 (19.2)	3.11 (3.10–3.13)	8273 (0.5)	1.10 (1.07–1.12)
**Number of CMDs**					
0	6 897 897 (28.2)	466 178 (6.8)	1.00 (Reference)	25 448 (0.4)	1.00 (Reference)
1	15 363 451 (62.8)	1 140 201 (7.4)	1.08 (1.08–1.09)	73 831 (0.5)	1.23 (1.21–1.24)
2	2 045 686 (8.4)	293 840 (14.4)	2.36 (2.35–2.37)	8943 (0.4)	1.16 (1.13–1.19)
3	140 835 (0.6)	32 497 (23.1)	4.30 (4.24–4.35)	669 (0.5)	1.26 (1.16–1.36)
Continuous			1.49 (1.48–1.49)		1.12 (1.11–1.13)
P for trend‡			<0.001		<0.001
**Patterns of CMDs**					
No CMDs	6 897 897 (28.2)	466 178 (6.8)	1.00 (Reference)	25 448 (0.4)	1.00 (Reference)
Only inadequate GWG	4 670 642 (19.1)	535 360 (11.5)	1.61 (1.61–1.62)	42 126 (0.9)	1.98 (1.95–2.01)
Only excessive GWG	9 930 398 (40.6)	497 019 (5.0)	0.74 (0.74–0.74)	28 614 (0.3)	0.79 (0.78–0.81)
Only GDM	428 047 (1.8)	39 227 (9.2)	1.51 (1.49–1.52)	1192 (0.3)	0.89 (0.84–0.94)
Only HDP	334 364 (1.4)	68 595 (20.5)	3.44 (3.41–3.48)	1899 (0.6)	1.33 (1.27–1.39)
Inadequate GWG + GDM	397 832 (1.6)	43 939 (11.0)	1.83 (1.81–1.85)	1654 (0.4)	1.28 (1.22–1.35)
Excessive GWG + GDM	551 180 (2.3)	48 948 (8.9)	1.41 (1.40–1.42)	1584 (0.3)	0.85 (0.80–0.89)
Inadequate GWG + HDP	211 783 (0.9)	55 439 (26.2)	4.35 (4.30–4.39)	2232 (1.1)	2.03 (1.95–2.12)
Excessive GWG + HDP	835 934 (3.4)	134 151 (16.1)	2.64 (2.63–2.66)	3290 (0.4)	0.98 (0.95–1.02)
GDM + HDP	48 957 (0.2)	11 363 (23.2)	4.35 (4.25–4.45)	183 (0.4)	1.02 (0.88–1.18)
Inadequate GWG + GDM + HDP	40 641 (0.2)	9858 (24.3)	4.57 (4.46–4.68)	247 (0.6)	1.60 (1.41–1.81)
Excessive GWG + GDM + HDP	100 194 (0.4)	22 639 (22.6)	4.13 (4.06–4.19)	422 (0.4)	1.10 (1.00–1.21)

CI – confidence interval, CMD – cardiometabolic disorder, GDM – gestational diabetes mellitus, GWG – gestational weight gain, HDP – hypertensive disorders during pregnancy, OR – odds ratio

*Models were adjusted for maternal age, race/ethnicity, marital status, educational level, parity, time of initiation of prenatal care, total number of prenatal care visits, smoking before or during pregnancy, infant sex.

†Three separate models were built for each individual CMDs.

‡Models were constructed by considering the total CMD number as a continuous variable.

**Figure 2:**
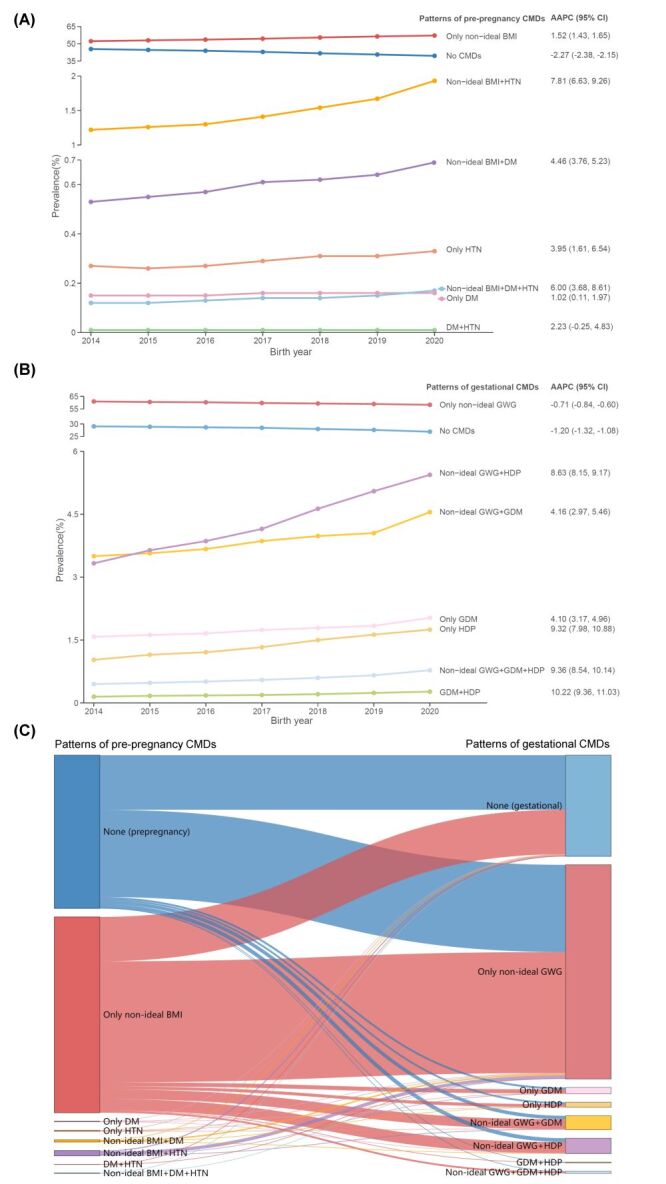
Trends and progression of pre-pregnancy and gestational cardiometabolic disorders patterns. **Panel A.** Trends of patterns of pre-pregnancy cardiometabolic disorders. **Panel B.** Trends of patterns of gestational cardiometabolic disorders. **Panel C.** Progression of patterns of pre-pregnancy cardiometabolic disorders to patterns of gestational cardiometabolic disorders. AAPC – average annual percent change, BMI – body mass index, CI – confidence interval, CMD – cardiometabolic disorder, DM – diabetes mellitus, HTN – hypertension, GDM – gestational diabetes mellitus, GWG – gestational weight gain, HDP – hypertensive disorders during pregnancy.

The rates of CMD patterns increased significantly from 2014 to 2020 with a positive AAPC, particularly for patterns with two or more CMDs, such as both non-ideal BMI and hypertension (AAPC 7.8%, 95% CI = 6.6–9.3%), and both GDM and HDP (AAPC 10.2%, 95% CI = 9.4–11.0%) (**Figure 2**, Panels A–B; Table S2 in the **Online Supplementary Document**). Of the women without pre-pregnancy CMDs, 35.6% had no gestational CMDs while 56.9% developed GWG (**Figure 2**, Panel C; Table S3 in the **Online Supplementary Document**). Most women with pre-pregnancy non-ideal BMI had either non-ideal GWG (61.8%) or no gestational CMDs (22.7%). Besides, all possible associations between pre-pregnancy CMDs and gestational CMDs were significant (Table S4 in the **Online Supplementary Document**).

### Associations of pre-pregnancy CMDs with preterm birth and infant mortality

All individual CMDs were associated with preterm birth and infant mortality (**Table 2**; Table S5 in the **Online Supplementary Document**). There were dose-response relationships between the number of pre-pregnancy CMDs and preterm birth as well as infant mortality (*P* for trend <0.001). For example, compared with no pre-pregnancy CMDs, the odds ratios of preterm birth were 1.17 (95% CI = 1.17–1.18) for one, 3.43 (95% CI = 3.40–3.45) for two, 6.99 (95% CI = 6.82–7.16) for three CMD(s), respectively (**Table 2**). All pre-pregnancy CMD patterns were associated with preterm birth and infant mortality (**Table 2**). The co-occurrence of diabetes and hypertension showed the strongest associations with preterm birth (OR = 10.52; 95% CI = 9.71–11.40) and infant mortality (OR = 3.93; 95% CI = 2.99–5.18) compared with no pre-pregnancy CMDs.

The associations between pre-pregnancy CMDs and subclassification of preterm birth (moderately, very, and extremely preterm birth) and infant mortality (neonatal and post-neonatal mortality) are consistent with the main results (Table S6 in the **Online Supplementary Document**). However, higher risks were observed for neonatal mortality than for post-neonatal mortality. For instance, the OR for the association between diabetes and neonatal mortality was 2.45 (95% CI = 2.33–2.58), while that for post-neonatal mortality was 2.11 (95% CI = 1.97–2.27). Similar results were observed in sensitivity analyses and subgroup analyses (Table S7–12 in the **Online Supplementary Document**).

### Associations of gestational CMDs with preterm birth and infant mortality

Individual gestational CMDs were positively associated with preterm birth and infant mortality, except for excessive GWG and GDM (**Table 3**; Table S5 in the **Online Supplementary Document**). There were dose-response associations of gestational CMD with preterm birth and infant mortality (*P* for trend <0.001). All gestational CMD patterns were associated with preterm birth, the coexistence of inadequate GWG, GDM and HDP showed the strongest association (OR = 4.57; 95% CI = 4.46–4.68). Of the 12 gestational CMD patterns, five of them were associated with a higher risk of infant mortality (**Table 3**).

Similar results were observed in the associations between most patterns of gestational CMDs with subclassification of preterm birth (moderately, very, and extremely preterm birth) and infant mortality (neonatal and post-neonatal mortality) (Table S6 in the **Online Supplementary Document**). Overall, higher risks were observed for neonatal mortality than for post-neonatal mortality across most gestational CMDs. However, there were exceptions; for instance, the odds ratio for the association of only HDP with neonatal mortality was 1.25 (95% CI = 1.17–1.33), while that for post-neonatal mortality was 1.47 (95% CI = 1.37–1.58), showing a higher risk for post-neonatal mortality in this specific case. Similar results were observed in sensitivity analyses and subgroup analyses (Table S7–12 in the **Online Supplementary Document**).

### Combinations of pre-pregnancy and gestational CMDs with preterm birth and infant mortality

All individual pre-pregnancy and gestational CMDs were associated with preterm birth and infant mortality (Table S13 in the **Online Supplementary Document**). Among the 108 combined patterns, the pattern with underweight BMI, inadequate GWG, diabetes, and HDP had the highest preterm birth (69.4%) and infant mortality (6.5%) (Table S14 in the **Online Supplementary Document**).

**Figure 3:**
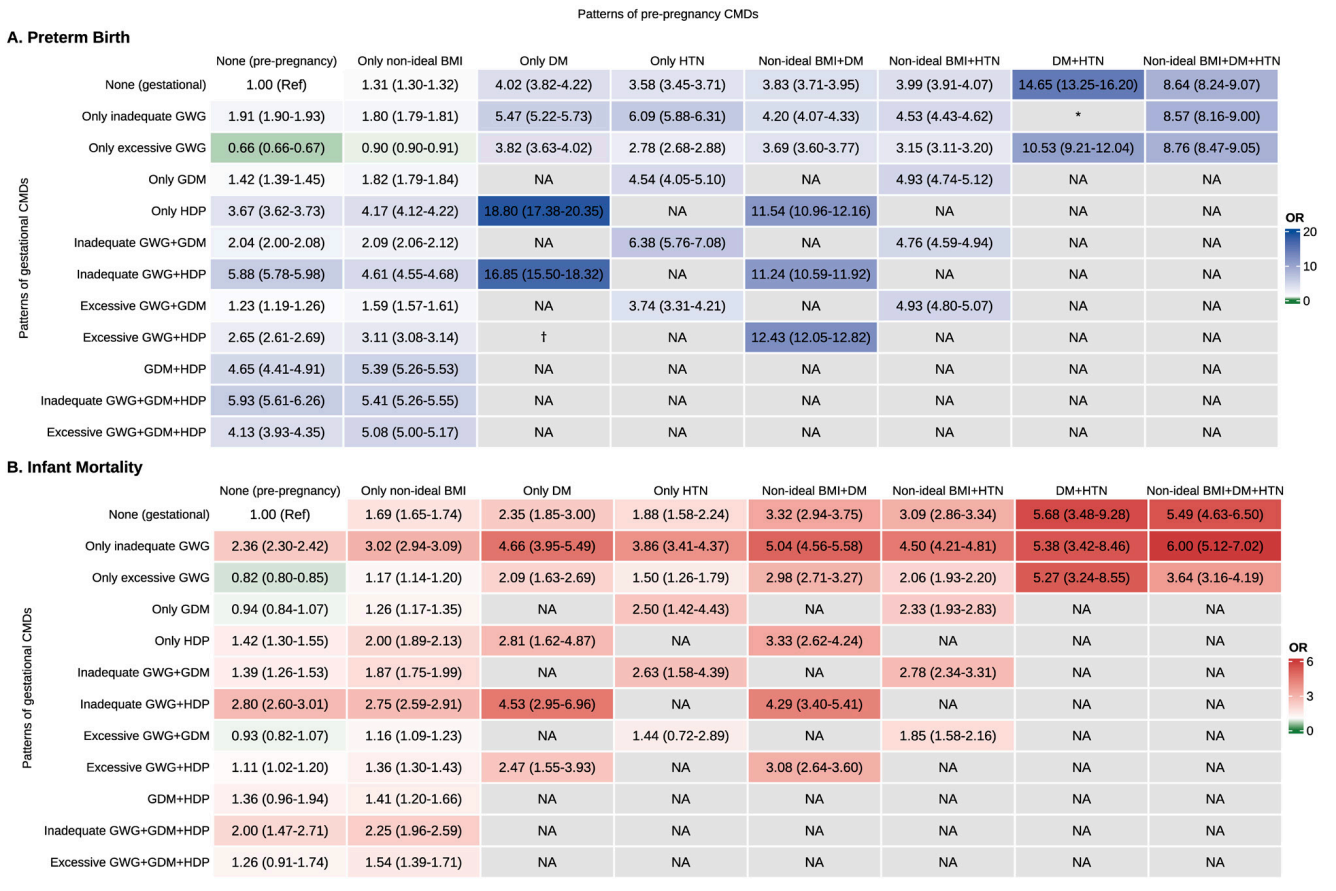
Associations of combinations of pre-pregnancy and gestational cardiometabolic disorders with preterm birth and infant mortality (n = 24 447 869). **Panel A.** Preterm birth. **Panel B.** Infant mortality. Data were presented as odds ratio (95% confidence interval). NA indicates non-existent patterns because pregnant women with pre-pregnancy diabetes cannot be diagnosed with gestational diabetes, and those with pre-pregnancy hypertension cannot have gestational hypertension. Models were adjusted for maternal age, race/ethnicity, marital status, educational level, parity, time of initiation of prenatal care, total number of prenatal care visits, smoking before or during pregnancy, infant sex. *For model stability and convergence, the patterns ‘DM, HTN, and inadequate GWG’ and ‘DM, HTN, and excessive GWG’ were combined into a single group. †For model stability and convergence, the patterns ‘DM, HDP, and inadequate GWG’ and ‘DM, HDP, and excessive GWG’ were combined into a single group. BMI – body mass index, CMD – cardiometabolic disorder, DM – diabetes mellitus, HTN – hypertension, GDM – gestational diabetes mellitus, GWG – gestational weight gain, HDP – hypertensive disorders during pregnancy.

Most combined patterns were associated with higher risks of preterm birth and infant mortality compared to no CMDs (**Figure 3**). Women with pre-pregnancy diabetes who developed HDP had an 18.80-fold increased odd of preterm birth (95% CI = 17.38–20.35). Women with both pre-pregnancy diabetes and hypertension experienced the highest risk of infant mortality (OR = 5.68; 95% CI = 3.48–9.28). Furthermore, when this risk pattern was combined with inadequate GWG and non-ideal BMI, the corresponding OR for infant mortality increased to 6.00 (95% CI = 5.12–7.02). The robustness of these findings was confirmed by Firth logistic regression analyses (Table S15 in the **Online Supplementary Document**).

## DISCUSSION

From 2014 to 2020, the prevalence of pre-pregnancy and gestational CMDs increased in the USA, and notably that of multiple co-occurring CMDs. Women with more CMDs experienced higher risks of preterm birth and infant mortality, particularly in those with both pre-pregnancy and gestational CMDs. Among pre-pregnancy CMD patterns, the co-occurrence of diabetes and hypertension showed the strongest association with preterm birth and infant mortality. Among gestational CMDs, women with inadequate GWG, GDM and HDP, or with inadequate GWG and HDP, had the highest risks of preterm birth and infant mortality, respectively. Additionally, women with pre-pregnancy diabetes combined with HDP experienced higher risks of preterm birth.

### Interpretation and comparison

Consistent with previous studies using the same data set [5,26,28], our findings confirmed increasing rates of individual CMDs and extended this evidence by demonstrating rising trends in multi-CMD patterns (≥2 CMDs). Several factors may explain these upward trends. The rising incidence of gestational diabetes and hypertension could be partially attributed to the utilisation of more stringent diagnostic criteria for these conditions, as well as institution-specific changes in screening and diagnostic protocols during the study period [29,30].

The observed associations of individual CMDs with preterm birth align with most previous studies [9,10,14,16,31], and associations of CMDs with infant mortality match previous studies on stillbirth, neonatal mortality, or perinatal mortality [11,12,15,32]. However, while prior studies focused mainly on single CMDs, our study comprehensively examined associations of individual, multiple, separate, and combined pre-pregnancy/gestational CMD patterns in one large nationwide data set, providing comparable effect estimates across CMDs and their co-occurrence. Although the large sample size enhances statistical power, this can also result in statistical significance even with minimal effect sizes. For example, the OR for infant mortality associated with maternal overweight was 1.07, indicating only marginal risk increases. While statistically significant, such findings may have limited clinical or public health impact. Distinguishing statistical significance from clinical meaningfulness is critical to translating these findings into practice.

The observed dose-response relationships between the number of CMDs and the risk of preterm birth and infant mortality suggest additive effects of multiple CMDs. This result aligns with previous observations that CMDs were often interrelated and each additional CMD was associated with adverse pregnancy outcomes [20,33]. Consequently, disease count could be an important predictive factor and a convenient method for predicting clinical outcomes among pregnant women during the peripartum period. These findings also emphasise the importance of interventions to prevent the onset of additional CMDs during the pregnancy among women with existing CMD.

Our findings show that patterns of pre-pregnancy CMDs (*e.g*. cooccurrence of pre-pregnancy diabetes and hypertension) were associated with increased risk of preterm birth confirm observations in previous studies. For example, a smaller Canadian population-based cohort study reported an OR of 6.34 (95% CI = 5.15–7.81) for this association [23]. Another study found that the concurrent presence of chronic hypertension and diabetes may have an additive effect on stillbirth [22]. This is biologically plausible, aligning with mechanisms (insulin resistance, oxidative stress, inflammation, mental stress [34]) underlying preterm birth and infant mortality in mothers with these co-occurring conditions. Notably, few studies have examined the effects of CMD patterns during pregnancy. Our work indicates these may further elevate adverse pregnancy outcome risk, with a stronger association for preterm birth than infant mortality. Future research should investigate the mechanisms underlying these associations.

Our study showed diabetic women who developed HDP face markedly heightened preterm birth risk. However, the observed extreme OR should be interpreted cautiously, as they may partly reflect residual confounding, misclassification, or limited sample size rather than true biological effects. Previous studies found a higher risk of preterm birth in pre-pregnancy diabetes compared to gestational diabetes [10,35], possibly due to the severity and prolonged nature of hyperglycaemia present in women with pre-pregnancy diabetes, which may worsen foetal development [36]. Furthermore, pregnancy introduces significant physiological changes which affect cardiovascular and metabolic systems [37], potentially challenging the perinatal health of women with pre-existing chronic conditions. For instance, pre-pregnancy diabetes increases the risk of preeclampsia, a major risk factor for preterm birth [4,38]. These mechanisms may partially explain the remarkable risk of co-occurring pre-pregnancy diabetes and HDP.

Our infant mortality subclassification analyses identified distinct associations between CMDs and infant mortality. Most CMDs, such as pre-pregnancy diabetes and hypertension, were associated with a higher risk of neonatal mortality than post-neonatal mortality, whereas HDP showed the opposite. However, the mechanisms underlying these differences remain to be elucidated. Neonatal mortality is more influenced by intrauterine insults (from obesity, diabetes, and hypertension), which cause early placental dysfunction and restricted foetal growth, ultimately leading to higher risks of stillbirth or early neonatal death [19]. In contrast, HDP, such as preeclampsia, which may enhance foetal lung maturity, lowering neonatal mortality, especially in preterm or small-for-gestational-age infants [39]. However, the protective effect diminishes in the post-neonatal period, where prematurity-related complications and long-term developmental issues become more significant.

### Implications

Our findings indicate that tailored prevention and management of multiple co-occurring CMDs in the peripartum period can lead to substantial individual, clinical, and public health benefits. At the individual level, early preconception counselling and health screenings are essential, particularly for women with pre-pregnancy CMDs [40]. Beyond regular prenatal care, women planning pregnancy could benefit from tailored interventions such as structured weight management, glucose/blood pressure monitoring, and nutritional counselling, which help stabilise existing CMDs and reduce the risk of developing additional conditions during pregnancy. At the clinical level, integrated prenatal management is needed, along with risk-stratified screening guidelines to identify high-risk women before and during pregnancy [41]. For instance, women with pre-pregnancy diabetes require earlier, more frequent blood pressure monitoring and timely treatment to prevent gestational hypertension or preeclampsia and preterm birth. For women with obesity, individualised weight gain targets and multidisciplinary management involving obstetricians, endocrinologists, and dietitians are recommended. At the public health level, efforts should focus on the combined burden of CMDs rather than isolated conditions. Policymakers could incorporate CMD risk assessment into routine preconception programmes, expand community-based services access, and provide lifestyle and weight management programmes for high-risk women. Insurance coverage and incentives for integrated prenatal care, along with public education campaigns, can further enhance prevention and management [42,43].

### Strength and limitations

A strength of this study is the comprehensive examination of both the number and the patterns of pre-pregnancy and gestational CMDs with preterm birth and infant mortality. Furthermore, the study including over 24 million mothers provided sufficient statistical power to examine these associations.

This study has several limitations. First, self-reported pre-pregnancy weight/height may introduce recall bias and misclassification of BMI and GWG. While existing evidence suggests that high consistency with measured values among US reproductive women [44], underreporting of weight – especially among women with higher body weight – could bias BMI downward and inflate GWG estimates. This is unlikely to alter category assignments but may attenuate associations with perinatal outcomes. Second, unplanned pregnancies and irregular prenatal visits, pregnancies with diabetes or hypertension may include misclassifications, potentially contributing to over- or underestimation of the associations with pregnancy outcomes. Third, the severity and management of CMDs are important factors influencing preterm birth and infant mortality. However, our data lacked information on treatment strategies and medication use. In addition, women with pre-pregnancy or gestational CMDs may receive intensified monitoring or interventions that influence preterm birth detection and management. Although we adjusted for prenatal visit frequency, unavailable details on health care utilisation introduce residual confounding by indication. Fourth, additional key covariates at both the individual and community levels were unavailable for adjustment. Individual-level factors include family history of diabetes/hypertension and modifiable lifestyles (*e.g*. diet, physical activity); community-level factors include socioeconomic status (*e.g*. household income, neighbourhood deprivation) and physical environment (*e.g*. prenatal care access). Thus, residual confounding from these unmeasured factors cannot be ruled out, potentially weakening the robustness of our findings. Fifth, the complexity introduced by multiple categorical variables makes it difficult to interpret subgroup-specific associations in combined CMD patterns. Moreover, while Firth logistic regression confirmed result stability, small sample sizes in some categories persist as a concern – introducing residual sparse data bias that may reduce estimate precision.

## CONCLUSIONS

The prevalence of multiple pre-pregnancy and gestational CMDs increased in the US from 2014 to 2020. This is likely to adversely affect pregnancy outcomes because women with multiple CMDs were found to experience elevated risks of preterm birth and infant mortality, compared to those with no or a single CMD. This underscores the need for a more comprehensive approach to addressing co-occurring CMDs at the pre-pregnancy and gestational periods. Improvements of the prevention and management of CMDs across before and during pregnancy are urgently needed.

## Additional material


Online Supplementary Document
