# Supplementary material for: Pre-pregnancy and gestational cardiometabolic disorders and risk of preterm birth and infant mortality
Source: J Glob Health. 2025 Dec 5;15:04333. doi: 10.7189/jogh.15.04333 (PMC12679054; doi:10.7189/jogh.15.04333)
Supplement: Online Supplementary Document [file jogh-15-04333-s001.pdf]

Supplement to: Cheng Y, Kivimäki M, Zhang Y, Carrillo-Larco R, Dai X, Wang Y, Xu X. Pre-pregnancy and gestational cardiometabolic disorders and risk of preterm birth and infant mortality. J Glob Health. 2025;15:04333.

Table S1. Comparison between included and excluded participants (N=26,986,716).
Table S2. The age-adjusted prevalence rates of pre-pregnancy and gestational cardiometabolic disorders from 2014 to 2020.
Table S3. Proportions of combined patterns of pre-pregnancy and gestational cardiometabolic disorders.
Table S4. Associations between pre-pregnancy and gestational CMDs.
Table S5. Associations of individual pre-pregnancy cardiometabolic disorders and gestational cardiometabolic disorders with preterm birth and infant mortality by additional adjusting each other.
Table S6. Associations of pre-pregnancy cardiometabolic disorders and gestational cardiometabolic disorders with the subtype of preterm birth and infant mortality.
Table S7. Associations of pre-pregnancy cardiometabolic disorders and gestational cardiometabolic disorders with preterm birth and infant mortality after excluding participants with incomplete covariate data.
Table S8. Associations of pre-pregnancy cardiometabolic disorders and gestational cardiometabolic disorders with preterm birth and infant mortality in primiparous women.
Table S9. Subgroup analysis for associations of pre-pregnancy cardiometabolic disorders and gestational cardiometabolic disorders with preterm birth and infant mortality by maternal age.
Table S10. Subgroup analysis for associations of pre-pregnancy cardiometabolic disorders and gestational cardiometabolic disorders with preterm birth and infant mortality by race/ethnicity.
Table S11. Subgroup analysis for associations of pre-pregnancy cardiometabolic disorders and gestational cardiometabolic disorders with preterm birth and infant mortality by marital status.
Table S12. Subgroup analysis for associations of pre-pregnancy cardiometabolic disorders and gestational cardiometabolic disorders with preterm birth and infant mortality by educational level.
Table S13. Associations of individual and number of pre-pregnancy and gestational cardiometabolic disorders with preterm birth and infant mortality.
Table S14. Comparison of the preterm birth and infant mortality between combinations of pre-pregnancy and gestational CMDs patterns.
Table S15. Associations of combinations of pre-pregnancy and gestational cardiometabolic disorders with preterm birth and infant mortality using Firth logistic regression.
S1 Checklist. STROBE Statement—Checklist of items that should be included in reports of cross-sectional studies.

Table S1. Comparison between included and excluded participants (N=26,986,716).

Characteristics	Included (n=24,447,869)	Excluded (n=2,538,847)	*P* value[a]	SMD[b]
**Maternal age (years)**			<0.001	0.461
<20	972,867 (4.0)	437,144 (17.2)		
20-24	5,032,357 (20.6)	375,408 (14.8)		
25-29	7,193,869 (29.4)	598,007 (23.6)		
30-34	6,989,754 (28.6)	659,271 (26.0)		
35-39	3,485,446 (14.3)	367,409 (14.5)		
⩾40	773,576 (3.2)	101,608 (4.0)		
**Race/ethnicity**			< 0.001	0.437
Non-Hispanic White	12,812,556 (52.4)	1,066,636 (42.0)		
Non-Hispanic Black	3,413,016 (14.0)	442,163 (17.4)		
Other	2,324,673 (9.5)	197,101 (7.8)		
Hispanic	5,702,836 (23.3)	590,545 (23.3)		
Unknown	194,788 (0.8)	242,402 (9.6)		
**Marital status**			< 0.001	0.184
Married	13,810,417 (56.5)	1,257,864 (49.5)		
Unmarried	8,967,019 (36.7)	1,153,960 (45.5)		
Unknown	1,670,433 (6.8)	127,023 (5.0)		
**Educational level**			< 0.001	0.553
Under high school	2,966,409 (12.1)	572,774 (22.6)		
High school	11,278,751 (46.1)	883,887 (34.8)		
Above high school	9,925,964 (40.6)	795,703 (31.3)		
Unknown	276,745 (1.1)	286,483 (11.3)		
**Parity**			< 0.001	0.142
Nulliparous	9,313,522 (38.1)	970,559 (38.2)		
Multiparous	15,079,343 (61.7)	1,529,100 (60.2)		
Unknown	55,004 (0.2)	39,188 (1.5)		
**Time of initiation of prenatal care**			< 0.001	0.610
1st to 3rd month	18,647,180 (76.3)	1,446,478 (52.6)		
4th to 6th month	3,921,111 (16.0)	394,556 (12.2)		
7th to final month	1,035,912 (4.2)	133,671 (4.1)		
No prenatal care	342,254 (1.4)	106,567 (4.0)		
Unknown	501,412 (2.1)	457,575 (27.1)		
**Total number of prenatal care visits**			< 0.001	0.594
0	342,253 (1.4)	106,567 (4.2)		
1-4 visits	850,074 (3.5)	143,605 (5.7)		
5-9 visits	5,030,394 (20.6)	518,911 (20.4)		
⩾10 visits	17,724,452 (72.5)	1,348,950 (53.1)		
Unknown	500,696 (2.1)	420,814 (16.6)		
**Smoking before pregnancy**			< 0.001	0.457
No	22,125,187 (90.5)	2,070,551 (81.6)		
Yes	2,185,714 (8.9)	191,064 (7.5)		

Unknown	136,968 (0.6)	277,232 (10.9)		
**Smoking during pregnancy**			< 0.001	0.458
No	22,634,813 (92.6)	2,109,101 (83.1)		
Yes	1,678,364 (6.9)	152,456 (6.0)		
Unknown	134,692 (0.6)	277,290 (10.9)		
**Infant sex**			< 0.001	0.006
Male	12,514,112 (51.2)	1,291,605 (50.9)		
Female	11,933,757 (48.8)	1,247,242 (49.1)		

SMD, standardized mean difference.

Values were expressed as number (%).

[a] χ^2 analyses were used to compare differences across groups.

[b] .2 is a small-effect size, .5 is a moderate-effect size, and .8 is a large-effect size.

Table S2. The age-adjusted prevalence rates of pre-pregnancy and gestational cardiometabolic disorders from 2014 to 2020.

CMDs	Year							AAPC (95% CI)
	**2014**	**2015**	**2016**	**2017**	**2018**	**2019**	**2020**	
**Pre-pregnancy CMDs**								
**Individual CMDs**								
Non-ideal BMI	54.13	54.91	55.62	56.6	57.78	58.86	59.95	1.71 (1.68, 1.76)
DM	0.81	0.83	0.86	0.91	0.93	0.96	1.03	4.05 (2.73, 5.48)
HTN	1.62	1.65	1.71	1.85	2.00	2.13	2.44	6.74 (6.11, 7.70)
**Number of CMDs**								
0	39.54	40.67	41.74	42.94	43.95	44.66	45.44	-2.27 (-2.38, -2.15)
1	52.68	53.4	54.04	54.89	55.94	56.86	57.66	1.53 (1.45, 1.64)
2	1.76	1.82	1.89	2.03	2.18	2.32	2.63	6.57 (5.96, 7.47)
3	0.12	0.12	0.13	0.14	0.14	0.15	0.17	6.00 (3.68, 8.61)
**Patterns of CMDs**								
No CMDs	39.54	40.67	41.74	42.94	43.95	44.66	45.44	-2.27 (-2.38, -2.15)
Only non-ideal BMI	52.26	52.99	53.62	54.44	55.47	56.4	57.17	1.52 (1.43, 1.65)
Only DM	0.15	0.15	0.15	0.16	0.16	0.16	0.16	1.02 (0.11, 1.97)
Only HTN	0.26	0.27	0.27	0.29	0.31	0.31	0.33	3.95 (1.61, 6.54)
Non-ideal BMI+DM	0.53	0.55	0.57	0.61	0.62	0.64	0.69	4.46 (3.76, 5.23)
Non-ideal BMI+HTN	1.22	1.26	1.3	1.41	1.54	1.67	1.93	7.81 (6.63, 9.26)
DM+HTN	0.01	0.01	0.01	0.01	0.01	0.01	0.01	2.23 (-0.25, 4.83)
Non-ideal BMI+DM+HTN	0.12	0.12	0.13	0.14	0.14	0.15	0.17	6.00 (3.68, 8.61)
**Gestational CMDs**								
**Individual CMDs**								
Non-ideal GWG	68.21	68.22	68.29	68.32	68.53	68.68	69.06	0.19 (0.14, 0.24)
GDM	5.68	5.84	6.02	6.34	6.58	6.79	7.63	4.86 (3.97, 5.65)
HDP	4.96	5.44	5.76	6.22	6.94	7.57	8.23	8.82 (8.33, 9.37)
**Number of CMDs**								
0	26.9	27.61	27.98	28.45	28.64	28.85	29.02	-1.20 (-1.32, -1.08)
1	62.07	62.39	62.6	62.8	63.15	63.29	63.55	-0.38 (-0.43, -0.34)
2	6.98	7.38	7.7	8.2	8.82	9.33	10.26	6.50 (6.15, 6.89)
3	0.45	0.48	0.51	0.55	0.6	0.66	0.78	9.36 (8.54, 10.14)
**Patterns of CMDs**								
No CMDs	26.9	27.61	27.98	28.45	28.64	28.85	29.02	-1.20 (-1.32, -1.08)
Only non-ideal GWG	58.3	58.92	59.32	59.72	60.28	60.52	60.94	-0.71 (-0.84, -0.60)
Only GDM	1.58	1.62	1.66	1.74	1.79	1.84	2.03	4.10 (3.17, 4.96)
Only HDP	1.03	1.15	1.21	1.33	1.5	1.63	1.75	9.32 (7.98, 10.88)
Non-ideal GWG+GDM	3.5	3.57	3.67	3.86	3.98	4.05	4.55	4.16 (2.97, 5.46)
Non-ideal GWG+HDP	3.33	3.64	3.86	4.15	4.63	5.05	5.44	8.63 (8.15, 9.17)
GDM+HDP	0.15	0.17	0.18	0.19	0.21	0.24	0.27	10.22 (9.36, 11.03)
Non-ideal GWG+GDM+HDP	0.45	0.48	0.51	0.55	0.6	0.66	0.78	9.36 (8.54, 10.14)

Prevalence rates per 100 mothers were age standardized, and they were weighted to account for all participants included in the analysis.

CI, confidence interval; AAPC, average annual percent change; CMD, cardiometabolic disorder; BMI, body mass index; DM, diabetes mellitus; HTN, hypertension; GWG, gestational weight gain; GDM, gestational diabetes mellitus; HDP, hypertensive disorders of pregnancy.

Table S3. Proportions of combined patterns of pre-pregnancy and gestational cardiometabolic disorders.

Patterns of pre-pregnancy CMDs	Patterns of gestational CMDs	Proportion (%)	Combine patterns Number (%)
None (pre-pregnancy)	None (gestational)	35.64%	3,723,376 (15.23%)
None (pre-pregnancy)	Only non-ideal GWG	56.90%	5,945,633 (24.32%)
None (pre-pregnancy)	Only GDM	1.28%	133,823 (0.55%)
None (pre-pregnancy)	Only HDP	1.17%	122,059 (0.50%)
None (pre-pregnancy)	Non-ideal GWG+GDM	2.25%	234,593 (0.96%)
None (pre-pregnancy)	Non-ideal GWG+HDP	2.52%	262,941 (1.08%)
None (pre-pregnancy)	GDM+HDP	0.08%	8,538 (0.03%)
None (pre-pregnancy)	Non-ideal GWG+GDM+HDP	0.17%	17,614 (0.07%)
Only non-ideal BMI	None (gestational)	22.71%	3,030,499 (12.40%)
Only non-ideal BMI	Only non-ideal GWG	61.84%	8,253,235 (33.76%)
Only non-ideal BMI	Only GDM	2.06%	275,230 (1.13%)
Only non-ideal BMI	Only HDP	1.53%	204,187 (0.84%)
Only non-ideal BMI	Non-ideal GWG+GDM	4.96%	661,977 (2.71%)
Only non-ideal BMI	Non-ideal GWG+HDP	5.68%	757,684 (3.10%)
Only non-ideal BMI	GDM+HDP	0.30%	40,419 (0.17%)
Only non-ideal BMI	Non-ideal GWG+GDM+HDP	0.92%	123,221 (0.50%)
Only DM	None (gestational)	28.97%	11,077 (0.05%)
Only DM	Only non-ideal GWG	57.52%	21,992 (0.09%)
Only DM	Only HDP	4.01%	1,535 (0.01%)
Only DM	Non-ideal GWG+HDP	9.50%	3,631 (0.01%)
Only HTN	None (gestational)	31.34%	22,284 (0.09%)
Only HTN	Only non-ideal GWG	60.59%	43,086 (0.18%)
Only HTN	Only GDM	2.66%	1,893 (0.01%)
Only HTN	Non-ideal GWG+GDM	5.40%	3,843 (0.02%)
Non-ideal BMI+DM	None (gestational)	20.73%	30,436 (0.12%)
Non-ideal BMI+DM	Only non-ideal GWG	58.80%	86,328 (0.35%)
Non-ideal BMI+DM	Only HDP	4.48%	6,583 (0.03%)
Non-ideal BMI+DM	Non-ideal GWG+HDP	15.98%	23,461 (0.10%)
Non-ideal BMI+HTN	None (gestational)	19.80%	71,425 (0.29%)
Non-ideal BMI+HTN	Only non-ideal GWG	61.99%	223,657 (0.91%)
Non-ideal BMI+HTN	Only GDM	4.74%	17,101 (0.07%)
Non-ideal BMI+HTN	Non-ideal GWG+GDM	13.47%	48,599 (0.20%)
DM+HTN	None (gestational)	30.46%	824 (0.003%)
DM+HTN	Only non-ideal GWG	69.54%	1,881 (0.01%)
Non-ideal BMI+DM+HTN	None (gestational)	24.02%	7,976 (0.03%)
Non-ideal BMI+DM+HTN	Only non-ideal GWG	75.98%	25,228 (0.10%)

CMD, cardiometabolic disorder; BMI, body mass index; DM, diabetes mellitus; HTN, hypertension; GWG, gestational weight gain; GDM, gestational diabetes mellitus; HDP, hypertensive disorders of pregnancy.

Table S4. Associations between pre-pregnancy and gestational CMDs.

Exposures	Outcomes					
	DM	HTN	GDM	HDP	Inadequate GWG	Excessive GWG
BMI						
Underweight	0.67 (0.64-0.70)	0.68 (0.65-0.70)	0.85 (0.84-0.86)	0.73 (0.72-0.74)	1.13 (1.12-1.13)	0.54 (0.54-0.54)
Normal weight	1.00 (Ref)	1.00 (Ref)	1.00 (Ref)	1.00 (Ref)	1.00 (Ref)	1.00 (Ref)
Overweight	1.93 (1.91-1.96)	2.14 (2.12-2.16)	1.76 (1.76-1.77)	1.74 (1.73-1.75)	0.72 (0.72-0.72)	2.43 (2.42-2.44)
Obese	4.63 (4.58-4.68)	5.97 (5.92-6.02)	3.31 (3.30-3.33)	3.13 (3.12-3.15)	1.23 (1.23-1.24)	2.41 (2.41-2.42)
DM	-	8.55 (8.45-8.66)	-	2.71 (2.68-2.74)	1.26 (1.25-1.28)	1.28 (1.26-1.29)
HTN	8.52 (8.42-8.63)	-	2.37 (2.35-2.39)	-	1.22 (1.21-1.23)	1.30 (1.29-1.31)
GDM	-	-	-	2.22 (2.20-2.23)	1.40 (1.40-1.41)	0.95 (0.95-0.95)
HDP	-	-	2.21 (2.20-2.22)	-	0.96 (0.96-0.97)	1.63 (1.62-1.64)

Data were represented as odds ratio (95% confidence interval).

All separate models were adjusted for maternal age, race/ethnicity, marital status, educational level, parity, time of initiation of prenatal care, smoking before pregnancy, infant sex.

BMI, body mass index; DM, diabetes mellitus; HTN, hypertension; GWG, gestational weight gain; GDM, gestational diabetes mellitus; HDP, hypertensive disorders of pregnancy; NA, not applicable.

Table S5. Associations between individual pre-pregnancy cardiometabolic disorders and gestational cardiometabolic disorders with preterm birth and infant mortality by additional adjusting each other.

**CMDs**	Preterm birth	Infant mortality
**Pre-pregnancy CMDs[a]**		
BMI		
Underweight	1.32 (1.31-1.33)	1.07 (1.03-1.10)
Normal weight	1.00 (Ref)	1.00 (Ref)
Overweight	1.05 (1.04-1.05)	1.12 (1.10-1.14)
Obese	1.23 (1.23-1.23)	1.40 (1.38-1.42)
DM	3.38 (3.35-3.42)	1.96 (1.88-2.05)
HTN	2.59 (2.57-2.61)	1.54 (1.49-1.59)
**Gestational CMDs[b]**		
GWG		
Inadequate	1.54 (1.53-1.54)	1.92 (1.89-1.95)
Adequate	1.00 (Ref)	1.00 (Ref)
Excessive	0.76 (0.76-0.76)	0.80 (0.79-0.81)
GDM	1.45 (1.44-1.46)	0.84 (0.81-0.86)
HDP	3.27 (3.25-3.28)	1.19 (1.17-1.22)

Data were represented as odds ratio (95% confidence interval).

Model includes all three pre-pregnancy CMDs in one model, adjusted for maternal age, race/ethnicity, marital status, educational level, parity, time of initiation of prenatal care, total number of prenatal care visits, smoking before or during pregnancy, infant sex.

[b] Model includes all three gestational CMDs in one model, adjusted for maternal age, race/ethnicity, marital status, educational level, parity, time of initiation of prenatal care, total number of prenatal care visits, smoking before or during pregnancy, infant sex.

CMD, cardiometabolic disorder; BMI, body mass index; DM, diabetes mellitus; HTN, hypertension; GWG, gestational weight gain; GDM, gestational diabetes mellitus; HDP, hypertensive disorders of pregnancy.

Table S6. Associations between pre-pregnancy cardiometabolic disorders and gestational cardiometabolic disorders with the subtype of preterm birth and infant mortality.

	Preterm birth (< 37 weeks' gestation)			Infant mortality (1 year)	
	**Moderately (32-36 weeks)**	**Very (28-31 weeks)**	**Extremely (20-27 weeks)**	**Neonatal (0-27 days)**	**Post-neonatal (28-364 days)**
**Pre-pregnancy CMDs**					
**Individual CMDs***					
BMI					
Underweight	1.32 (1.31-1.33)	1.33 (1.30-1.37)	1.21 (1.17-1.25)	1.07 (1.02-1.12)	1.06 (1.01-1.12)
Normal weight	1.00 (Ref)	1.00 (Ref)	1.00 (Ref)	1.00 (Ref)	1.00 (Ref)
Overweight	1.05 (1.04-1.05)	1.11 (1.10-1.12)	1.25 (1.23-1.26)	1.17 (1.14-1.19)	1.06 (1.04-1.09)
Obese	1.28 (1.27-1.28)	1.48 (1.46-1.50)	1.85 (1.83-1.88)	1.53 (1.50-1.56)	1.30 (1.27-1.33)
DM	4.11 (4.07-4.16)	4.78 (4.64-4.92)	3.67 (3.53-3.83)	2.45 (2.33-2.58)	2.11 (1.97-2.27)
HTN	2.88 (2.86-2.91)	4.39 (4.30-4.47)	3.78 (3.68-3.88)	1.83 (1.76-1.91)	1.74 (1.66-1.84)
**Number of CMDs**					
0	1.00 (Ref)	1.00 (Ref)	1.00 (Ref)	1.00 (Ref)	1.00 (Ref)
1	1.15 (1.14-1.15)	1.27 (1.26-1.28)	1.49 (1.47-1.51)	1.31 (1.29-1.33)	1.16 (1.14-1.19)
2	3.27 (3.25-3.30)	4.73 (4.63-4.83)	4.63 (4.51-4.76)	2.33 (2.24-2.42)	1.99 (1.89-2.09)
3	6.62 (6.45-6.78)	10.65 (10.05-11.28)	9.88 (9.14-10.68)	3.78 (3.39-4.21)	2.84 (2.44-3.32)
**Pattern of CMDs**					
No CMDs	1.00 (Ref)	1.00 (Ref)	1.00 (Ref)	1.00 (Ref)	1.00 (Ref)
Only underweight	1.31 (1.31-1.31)	1.28 (1.28-1.28)	1.08 (1.08-1.08)	1.07 (1.02-1.11)	1.07 (1.01-1.12)
Only overweight	1.02 (1.02-1.02)	1.04 (1.04-1.04)	1.06 (1.06-1.06)	1.16 (1.13-1.18)	1.06 (1.03-1.08)
Only obese	1.19 (1.19-1.19)	1.29 (1.29-1.29)	1.35 (1.35-1.35)	1.49 (1.46-1.52)	1.27 (1.24-1.30)
Only DM	4.52 (4.52-4.52)	5.13 (5.12-5.14)	2.37 (2.37-2.38)	2.42 (2.11-2.77)	2.12 (1.77-2.54)
Only HTN	2.97 (2.97-2.97)	5.91 (5.91-5.92)	2.74 (2.73-2.74)	1.69 (1.52-1.88)	1.75 (1.53-2.00)

	Preterm birth (< 37 weeks' gestation)			Infant mortality (1 year)	
	**Moderately (32-36 weeks)**	**Very (28-31 weeks)**	**Extremely (20-27 weeks)**	**Neonatal (0-27 days)**	**Post-neonatal (28-364 days)**
Underweight + DM	4.75 (4.74-4.76)	7.86 (7.82-7.91)	2.69 (2.67-2.72)	2.86 (1.74-4.72)	1.93 (0.92-4.07)
Overweight + DM	4.24 (4.24-4.24)	4.84 (4.83-4.85)	2.34 (2.33-2.34)	2.41 (2.13-2.74)	2.11 (1.78-2.49)
Obese + DM	4.02 (4.02-4.02)	4.00 (3.99-4.00)	2.85 (2.85-2.86)	3.03 (2.81-3.27)	2.36 (2.13-2.63)
Underweight + HTN	3.79 (3.78-3.80)	11.43 (11.39-11.47)	2.99 (2.97-3.01)	2.84 (2.02-4.01)	1.67 (0.97-2.88)
Overweight + HTN	2.88 (2.88-2.88)	5.23 (5.23-5.24)	3.21 (3.20-3.21)	2.05 (1.88-2.24)	1.83 (1.63-2.06)
Obese + HTN	2.89 (2.89-2.89)	4.67 (4.67-4.68)	3.58 (3.58-3.58)	2.17 (2.06-2.29)	1.91 (1.78-2.04)
DM + HTN	0.67 (0.66-0.67)	3.41 (3.39-3.43)	0.49 (0.48-0.49)	4.46 (3.21-6.19)	3.14 (1.92-5.15)
Underweight + DM + HTN	0.00 (0.00-I)	0.00 (0.00-I)	0.00 (0.00-I)	2.36 (0.32-17.53)	0.01 (0.00-45E11)
Overweight + DM + HTN	5.65 (5.64-5.66)	24.97 (24.92-25.03)	7.39 (7.36-7.42)	3.56 (2.73-4.63)	3.28 (2.30-4.68)
Obese + DM + HTN	6.16 (6.16-6.17)	13.42 (13.41-13.44)	6.88 (6.87-6.90)	3.88 (3.44-4.37)	2.79 (2.35-3.32)
**Gestational CMDs**					
**Individual CMDs**					
GWG					
Inadequate	1.40 (1.40-1.41)	2.02 (1.99-2.04)	3.14 (3.10-3.19)	2.30 (2.25-2.34)	1.37 (1.34-1.41)
Adequate	1.00 (Ref)	1.00 (Ref)	1.00 (Ref)	1.00 (Ref)	1.00 (Ref)
Excessive	0.83 (0.83-0.84)	0.65 (0.64-0.66)	0.48 (0.47-0.49)	0.71 (0.70-0.73)	0.93 (0.91-0.95)
GDM	1.71 (1.70-1.72)	1.43 (1.41-1.46)	0.75 (0.73-0.78)	0.79 (0.76-0.82)	1.02 (0.97-1.06)
HDP	3.12 (3.10-3.13)	4.03 (3.98-4.08)	1.91 (1.87-1.95)	0.97 (0.94-0.99)	1.33 (1.29-1.38)
**Number of CMDs**					
0	1.00 (Ref)	1.00 (Ref)	1.00 (Ref)	1.00 (Ref)	1.00 (Ref)
1	1.05 (1.05-1.05)	1.23 (1.21-1.24)	1.48 (1.45-1.50)	1.31 (1.29-1.34)	1.10 (1.07-1.12)
2	2.33 (2.32-2.34)	3.02 (2.97-3.07)	1.81 (1.77-1.85)	1.09 (1.06-1.13)	1.26 (1.22-1.31)
3	4.41 (4.35-4.47)	4.04 (3.86-4.22)	1.65 (1.52-1.79)	1.11 (1.00-1.23)	1.48 (1.32-1.66)

	Preterm birth (< 37 weeks' gestation)			Infant mortality (1 year)	
	**Moderately (32-36 weeks)**	**Very (28-31 weeks)**	**Extremely (20-27 weeks)**	**Neonatal (0-27 days)**	**Post-neonatal (28-364 days)**
**Pattern of CMDs**					
No CMDs	1.00 (Ref)	1.00 (Ref)	1.00 (Ref)	1.00 (Ref)	1.00 (Ref)
Only inadequate GWG	1.46 (1.46-1.47)	2.20 (2.17-2.23)	3.34 (3.29-3.40)	2.38 (2.34-2.43)	1.39 (1.36-1.43)
Only excessive GWG	0.77 (0.76-0.77)	0.59 (0.58-0.60)	0.45 (0.44-0.46)	0.70 (0.68-0.72)	0.92 (0.90-0.95)
Only GDM	1.52 (1.51-1.54)	1.47 (1.41-1.53)	0.79 (0.74-0.85)	0.83 (0.77-0.90)	0.98 (0.90-1.07)
Only HDP	3.31 (3.27-3.34)	5.37 (5.24-5.50)	2.87 (2.77-2.99)	1.25 (1.17-1.33)	1.47 (1.37-1.58)
Inadequate GWG + GDM	1.77 (1.75-1.79)	2.27 (2.19-2.35)	1.81 (1.72-1.90)	1.36 (1.28-1.45)	1.17 (1.08-1.27)
Excessive GWG + GDM	1.47 (1.45-1.48)	1.03 (0.99-1.07)	0.43 (0.40-0.46)	0.72 (0.67-0.78)	1.02 (0.95-1.09)
Inadequate GWG + HDP	3.90 (3.85-3.94)	8.19 (7.99-8.40)	6.25 (6.05-6.47)	2.10 (1.99-2.22)	1.93 (1.80-2.08)
Excessive GWG + HDP	2.67 (2.66-2.69)	3.07 (3.00-3.13)	1.30 (1.25-1.35)	0.84 (0.80-0.88)	1.22 (1.16-1.28)
GDM + HDP	4.42 (4.32-4.52)	4.47 (4.17-4.79)	1.73 (1.51-1.99)	0.84 (0.69-1.03)	1.31 (1.06-1.61)
Inadequate GWG + GDM + HDP	4.45 (4.34-4.56)	6.08 (5.69-6.50)	3.18 (2.84-3.56)	1.56 (1.33-1.83)	1.67 (1.37-2.04)
Excessive GWG + GDM + HDP	4.31 (4.24-4.38)	3.05 (2.88-3.23)	0.94 (0.83-1.06)	0.91 (0.79-1.04)	1.40 (1.22-1.61)

Values were expressed as odds ratio (95% confidence interval). Models were adjusted for maternal age, race/ethnicity, marital status, educational level, parity, time of initiation of prenatal care, total number of prenatal care visits, smoking before or during pregnancy, infant sex.

*Separate models were built for each individual CMDs.

CMD, cardiometabolic disorder; BMI, body mass index; DM, diabetes mellitus; HTN, hypertension; GWG, gestational weight gain; GDM, gestational diabetes mellitus; HDP, hypertensive disorders of pregnancy.

Table S7. Associations between pre-pregnancy cardiometabolic disorders and gestational cardiometabolic disorders with preterm birth and infant mortality after excluding participants with incomplete covariate data.

	Preterm birth	Infant mortality
**Pre-pregnancy CMDs**		
**Individual CMDs***		
BMI		
Underweight	1.32 (1.31-1.33)	1.08 (1.04-1.12)
Normal weight	1.00 (Ref)	1.00 (Ref)
Overweight	1.06 (1.06-1.07)	1.12 (1.10-1.14)
Obese	1.33 (1.32-1.33)	1.43 (1.41-1.46)
DM	4.22 (4.18-4.27)	2.35 (2.25-2.46)
HTN	3.03 (3.00-3.05)	1.79 (1.73-1.85)
**Number of CMDs**		
0	1.00 (Ref)	1.00 (Ref)
1	1.17 (1.17-1.17)	1.25 (1.23-1.27)
2	3.44 (3.41-3.47)	2.18 (2.11-2.26)
3	7.05 (6.88-7.23)	3.45 (3.14-3.80)
**Patterns of CMDs**		
No CMDs	1.00 (Ref)	1.00 (Ref)
Only underweight	1.33 (1.32-1.34)	1.08 (1.04-1.12)
Only overweight	1.04 (1.04-1.05)	1.11 (1.10-1.13)
Only obese	1.24 (1.24-1.25)	1.40 (1.38-1.42)
Only DM	4.73 (4.61-4.86)	2.40 (2.13-2.69)
Only HTN	3.18 (3.12-3.25)	1.72 (1.57-1.88)
Underweight + DM	5.11 (4.56-5.73)	2.51 (1.58-3.96)
Overweight + DM	4.45 (4.34-4.56)	2.35 (2.11-2.62)
Obese + DM	4.16 (4.09-4.23)	2.75 (2.57-2.94)
Underweight + HTN	4.06 (3.73-4.43)	2.10 (1.51-2.93)
Overweight + HTN	3.09 (3.04-3.15)	1.94 (1.79-2.09)
Obese + HTN	3.11 (3.08-3.15)	2.06 (1.97-2.16)
DM + HTN	10.67 (9.80-11.61)	4.15 (3.12-5.53)
Underweight + DM + HTN	10.90 (7.12-16.69)	2.15 (0.29-15.62)
Overweight + DM + HTN	8.37 (7.87-8.90)	3.38 (2.67-4.27)
Obese + DM + HTN	6.84 (6.65-7.03)	3.51 (3.16-3.89)
**Gestational CMDs**		
**Individual CMDs**		
GWG		
Inadequate	1.53 (1.52-1.53)	1.88 (1.85-1.91)
Adequate	1.00 (Ref)	1.00 (Ref)
Excessive	0.81 (0.81-0.81)	0.81 (0.80-0.83)
GDM	1.66 (1.65-1.67)	0.88 (0.86-0.91)
HDP	3.11 (3.10-3.13)	1.10 (1.07-1.13)
**Number of CMDs**		

0	1.00 (Ref)	1.00 (Ref)
1	1.08 (1.07-1.08)	1.21 (1.20-1.23)
2	2.37 (2.36-2.38)	1.15 (1.12-1.18)
3	4.34 (4.28-4.40)	1.30 (1.20-1.41)
**Patterns of CMDs**		
No CMDs	1.00 (Ref)	1.00 (Ref)
Only inadequate GWG	1.60 (1.60-1.61)	1.94 (1.91-1.97)
Only excessive GWG	0.74 (0.74-0.74)	0.80 (0.79-0.82)
Only GDM	1.52 (1.50-1.53)	0.88 (0.82-0.94)
Only HDP	3.42 (3.39-3.46)	1.32 (1.26-1.39)
Inadequate GWG + GDM	1.84 (1.82-1.86)	1.28 (1.22-1.36)
Excessive GWG + GDM	1.42 (1.41-1.44)	0.85 (0.80-0.89)
Inadequate GWG + HDP	4.29 (4.24-4.34)	1.98 (1.89-2.07)
Excessive GWG + HDP	2.65 (2.63-2.67)	0.99 (0.95-1.03)
GDM + HDP	4.38 (4.27-4.48)	1.02 (0.87-1.19)
Inadequate GWG + GDM + HDP	4.66 (4.54-4.78)	1.69 (1.48-1.93)
Excessive GWG + GDM + HDP	4.15 (4.08-4.22)	1.13 (1.02-1.25)

Values were expressed as odds ratio (95% confidence interval).

[*] Separate models were built for each individual CMDs.

Models were adjusted for maternal age, race/ethnicity, marital status, educational level, parity, time of initiation of prenatal care, total number of prenatal care visits, smoking before or during pregnancy, infant sex.

CMD, cardiometabolic disorder; BMI, body mass index; DM, diabetes mellitus; HTN, hypertension; GWG, gestational weight gain; GDM, gestational diabetes mellitus; HDP, hypertensive disorders of pregnancy.

Table S8. Associations between pre-pregnancy cardiometabolic disorders and gestational cardiometabolic disorders with preterm birth and infant mortality in primiparous women.

	Preterm birth	Infant mortality
**Pre-pregnancy CMDs**		
**Individual CMDs***		
BMI		
Underweight	1.18 (1.17-1.20)	1.00 (0.95-1.05)
Normal weight	1.00 (Ref)	1.00 (Ref)
Overweight	1.15 (1.14-1.15)	1.22 (1.19-1.25)
Obese	1.55 (1.54-1.56)	1.75 (1.71-1.79)
DM	4.66 (4.58-4.75)	2.25 (2.10-2.42)
HTN	3.06 (3.02-3.10)	1.92 (1.83-2.03)
**Number of CMDs**		
0	1.00 (Ref)	1.00 (Ref)
1	1.29 (1.29-1.30)	1.41 (1.38-1.44)
2	3.80 (3.75-3.85)	2.52 (2.39-2.66)
3	7.89 (7.57-8.23)	3.49 (2.99-4.07)
**Patterns of CMDs**		
No CMDs	1.00 (Ref)	1.00 (Ref)
Only underweight	1.19 (1.18-1.21)	0.99 (0.94-1.05)
Only overweight	1.12 (1.12-1.13)	1.21 (1.18-1.24)
Only obese	1.46 (1.45-1.47)	1.71 (1.67-1.75)
Only DM	5.30 (5.09-5.51)	2.32 (1.95-2.77)
Only HTN	3.07 (2.98-3.17)	1.74 (1.50-2.01)
Underweight + DM	5.31 (4.51-6.25)	3.45 (1.97-6.07)
Overweight + DM	5.37 (5.17-5.58)	2.40 (2.02-2.86)
Obese + DM	4.86 (4.73-5.00)	2.96 (2.66-3.30)
Underweight + HTN	3.91 (3.43-4.45)	2.79 (1.78-4.38)
Overweight + HTN	3.24 (3.15-3.34)	2.07 (1.83-2.35)
Obese + HTN	3.40 (3.34-3.46)	2.57 (2.40-2.76)
DM + HTN	12.23 (10.69-13.99)	3.18 (1.85-5.46)
Underweight + DM + HTN	10.07 (5.13-19.76)	0.01 (0.00-93E16)
Overweight + DM + HTN	9.85 (8.91-10.90)	3.98 (2.79-5.69)
Obese + DM + HTN	7.61 (7.27-7.97)	3.47 (2.92-4.11)
**Gestational CMDs**		
**Individual CMDs**		
GWG		
Inadequate	1.58 (1.57-1.59)	2.26 (2.20-2.31)
Adequate	1.00 (Ref)	1.00 (Ref)
Excessive	0.80 (0.79-0.80)	0.74 (0.72-0.76)
GDM	1.66 (1.65-1.68)	0.82 (0.78-0.87)
HDP	2.99 (2.97-3.01)	0.95 (0.92-0.99)
**Number of CMDs**		
0	1.00 (Ref)	1.00 (Ref)

1	1.06 (1.05-1.06)	1.24 (1.21-1.27)
2	2.39 (2.37-2.41)	1.08 (1.04-1.12)
3	4.20 (4.11-4.29)	1.12 (0.98-1.27)
**Patterns of CMDs**		
No CMDs	1.00 (Ref)	1.00 (Ref)
Only inadequate GWG	1.67 (1.66-1.68)	2.33 (2.27-2.39)
Only excessive GWG	0.72 (0.72-0.73)	0.73 (0.71-0.75)
Only GDM	1.48 (1.45-1.51)	0.78 (0.70-0.88)
Only HDP	3.35 (3.30-3.40)	1.17 (1.09-1.26)
Inadequate GWG + GDM	1.86 (1.83-1.90)	1.42 (1.30-1.55)
Excessive GWG + GDM	1.36 (1.33-1.38)	0.75 (0.68-0.82)
Inadequate GWG + HDP	4.44 (4.37-4.52)	2.04 (1.91-2.18)
Excessive GWG + HDP	2.48 (2.45-2.50)	0.86 (0.82-0.91)
GDM + HDP	4.29 (4.14-4.44)	0.85 (0.66-1.10)
Inadequate GWG + GDM + HDP	4.65 (4.47-4.84)	1.67 (1.36-2.05)
Excessive GWG + GDM + HDP	3.99 (3.89-4.08)	0.92 (0.78-1.07)

Values were expressed as odds ratio (95% confidence interval).

* Separate models were built for each individual CMDs.

Models were adjusted for maternal age, race/ethnicity, marital status, educational level, parity, time of initiation of prenatal care, total number of prenatal care visits, smoking before or during pregnancy, infant sex.

CMD, cardiometabolic disorder; BMI, body mass index; DM, diabetes mellitus; HTN, hypertension; GWG, gestational weight gain; GDM, gestational diabetes mellitus; HDP, hypertensive disorders of pregnancy.

Table S9. Subgroup analysis for associations between pre-pregnancy cardiometabolic disorders and gestational cardiometabolic disorders with preterm birth and infant mortality by maternal age.

	Preterm birth		Infant mortality	
**Age**	<35 years	≥35 years	<35 years	≥35 years
**Pre-pregnancy CMDs**				
**Individual CMDs***				
BMI				
Underweight	1.30 (1.29-1.31)	1.17 (1.14-1.20)	1.08 (1.04-1.12)	0.95 (0.84-1.06)
Normal weight	1.00 (Ref)	1.00 (Ref)	1.00 (Ref)	1.00 (Ref)
Overweight	1.04 (1.04-1.05)	1.18 (1.17-1.20)	1.11 (1.09-1.13)	1.19 (1.14-1.23)
Obese	1.28 (1.27-1.28)	1.56 (1.55-1.57)	1.41 (1.39-1.43)	1.51 (1.46-1.57)
DM	4.65 (4.60-4.71)	3.35 (3.28-3.41)	2.41 (2.29-2.53)	2.11 (1.95-2.28)
HTN	3.15 (3.12-3.18)	2.94 (2.90-2.98)	1.81 (1.74-1.88)	1.77 (1.68-1.88)
**Number of CMDs**				
0	1.00 (Ref)	1.00 (Ref)	1.00 (Ref)	1.00 (Ref)
1	1.15 (1.14-1.15)	1.31 (1.30-1.32)	1.24 (1.22-1.25)	1.30 (1.26-1.35)
2	3.59 (3.56-3.63)	3.39 (3.34-3.43)	2.19 (2.12-2.28)	2.18 (2.06-2.32)
3	7.97 (7.72-8.22)	6.50 (6.27-6.74)	3.66 (3.27-4.10)	3.14 (2.72-3.63)
**Patterns of CMDs**				
No CMDs	1.00 (Ref)	1.00 (Ref)	1.00 (Ref)	1.00 (Ref)
Only underweight	1.32 (1.31-1.33)	1.19 (1.16-1.22)	1.08 (1.05-1.12)	0.95 (0.85-1.07)
Only overweight	1.02 (1.02-1.03)	1.15 (1.14-1.16)	1.10 (1.08-1.12)	1.17 (1.13-1.22)
Only obese	1.21 (1.20-1.21)	1.44 (1.43-1.46)	1.38 (1.36-1.40)	1.45 (1.39-1.50)
Only DM	5.16 (5.02-5.31)	3.14 (2.97-3.32)	2.44 (2.17-2.75)	1.69 (1.28-2.22)
Only HTN	3.23 (3.15-3.30)	3.33 (3.22-3.45)	1.68 (1.52-1.86)	1.81 (1.55-2.11)
Underweight + DM	5.32 (4.74-5.97)	2.91 (2.18-3.88)	2.70 (1.75-4.18)	1.47 (0.36-5.97)
Overweight + DM	4.85 (4.72-4.98)	3.45 (3.30-3.60)	2.34 (2.08-2.64)	2.12 (1.74-2.58)
Obese + DM	4.40 (4.32-4.48)	3.76 (3.65-3.86)	2.77 (2.57-2.98)	2.66 (2.36-2.99)
Underweight + HTN	4.16 (3.80-4.55)	3.85 (3.22-4.61)	2.46 (1.79-3.37)	1.96 (0.92-4.16)
Overweight + HTN	3.15 (3.08-3.21)	3.29 (3.19-3.38)	1.98 (1.82-2.16)	1.93 (1.70-2.18)
Obese + HTN	3.17 (3.13-3.21)	3.25 (3.19-3.31)	2.02 (1.92-2.13)	2.12 (1.96-2.30)
DM + HTN	12.46 (11.29-13.74)	8.20 (7.12-9.43)	3.55 (2.50-5.04)	4.78 (3.06-7.48)
Underweight + DM + HTN	10.78 (6.83-17.03)	7.42 (3.20-17.25)	1.83 (0.25-13.49)	0.01 (0.00-29E27)
Overweight + DM + HTN	9.63 (8.92-10.40)	7.55 (6.91-8.25)	4.07 (3.14-5.27)	2.65 (1.81-3.87)
Obese + DM + HTN	7.63 (7.37-7.89)	6.33 (6.08-6.59)	3.60 (3.17-4.09)	3.27 (2.79-3.82)
**Gestational CMDs**				
**Individual CMDs**				
GWG				
Inadequate	1.55 (1.54-1.56)	1.42 (1.41-1.43)	1.93 (1.90-1.96)	1.82 (1.76-1.89)
Adequate	1.00 (Ref)	1.00 (Ref)	1.00 (Ref)	1.00 (Ref)
Excessive	0.79 (0.79-0.79)	0.88 (0.87-0.88)	0.80 (0.79-0.82)	0.81 (0.78-0.84)
GDM	1.72 (1.71-1.74)	1.57 (1.56-1.59)	0.89 (0.86-0.92)	0.85 (0.81-0.89)
HDP	3.12 (3.11-3.14)	3.15 (3.12-3.18)	1.08 (1.05-1.10)	1.18 (1.12-1.24)
**Number of CMDs**				

0	1.00 (Ref)	1.00 (Ref)	1.00 (Ref)	1.00 (Ref)
1	1.07 (1.07-1.08)	1.12 (1.11-1.13)	1.23 (1.21-1.25)	1.20 (1.16-1.24)
2	2.44 (2.42-2.45)	2.22 (2.19-2.24)	1.16 (1.12-1.19)	1.16 (1.10-1.22)
3	4.47 (4.40-4.55)	4.14 (4.04-4.24)	1.30 (1.18-1.42)	1.15 (0.99-1.33) [c]
**Patterns of CMDs**				
No CMDs	1.00 (Ref)	1.00 (Ref)	1.00 (Ref)	1.00 (Ref)
Only inadequate GWG	1.62 (1.62-1.63)	1.50 (1.49-1.52)	1.99 (1.95-2.02)	1.93 (1.85-2.00)
Only excessive GWG	0.72 (0.72-0.72)	0.82 (0.81-0.82)	0.79 (0.78-0.81)	0.79 (0.76-0.82)
Only GDM	1.56 (1.54-1.58)	1.46 (1.43-1.49)	0.90 (0.84-0.96)	0.86 (0.77-0.95)
Only HDP	3.43 (3.40-3.47)	3.49 (3.42-3.57)	1.30 (1.23-1.37)	1.43 (1.29-1.58)
Inadequate GWG + GDM	1.91 (1.89-1.94)	1.73 (1.70-1.76)	1.29 (1.22-1.37)	1.23 (1.12-1.35)
Excessive GWG + GDM	1.43 (1.41-1.45)	1.40 (1.37-1.43)	0.85 (0.80-0.91)	0.80 (0.73-0.89)
Inadequate GWG + HDP	4.36 (4.31-4.41)	4.23 (4.13-4.33)	2.04 (1.94-2.14)	1.99 (1.80-2.21)
Excessive GWG + HDP	2.61 (2.59-2.63)	2.79 (2.75-2.83)	0.95 (0.92-0.99)	1.11 (1.02-1.21)
GDM + HDP	4.59 (4.46-4.71)	4.10 (3.94-4.25)	0.99 (0.82-1.18)	1.07 (0.84-1.36)
Inadequate GWG + GDM + HDP	4.75 (4.61-4.89)	4.37 (4.19-4.55)	1.71 (1.48-1.99)	1.35 (1.07-1.70)
Excessive GWG + GDM + HDP	4.25 (4.17-4.33)	3.97 (3.86-4.09)	1.11 (0.99-1.24)	1.04 (0.87-1.26)

Values were expressed as odds ratio (95% confidence interval).

[*] Separate models were built for each individual CMDs.

Models were adjusted for race/ethnicity, marital status, educational level, parity, time of initiation of prenatal care, total number of prenatal care visits, smoking before or during pregnancy, infant sex.

CMD, cardiometabolic disorder; BMI, body mass index; DM, diabetes mellitus; HTN, hypertension; GWG, gestational weight gain; GDM, gestational diabetes mellitus; HDP, hypertensive disorders of pregnancy.

Table S10. Subgroup analysis for associations between pre-pregnancy cardiometabolic disorders and gestational cardiometabolic disorders with preterm birth and infant mortality by race/ethnicity.

	Preterm birth		Infant mortality	
Race/ethnicity	Non-Hispanic White	The others	Non-Hispanic White	The others
**Pre-pregnancy CMDs**				
**Individual CMDs***				
Non-ideal BMI				
Underweight	1.39 (1.37-1.40)	1.23 (1.22-1.25)	1.09 (1.04-1.14)	1.03 (0.98-1.08)
Normal weight	1.00 (Ref)	1.00 (Ref)	1.00 (Ref)	1.00 (Ref)
Overweight	1.07 (1.06-1.07)	1.06 (1.05-1.06)	1.11 (1.09-1.14)	1.14 (1.12-1.17)
Obese	1.38 (1.38-1.39)	1.30 (1.29-1.31)	1.41 (1.38-1.44)	1.52 (1.49-1.55)
DM	4.99 (4.92-5.07)	3.60 (3.55-3.65)	2.36 (2.20-2.53)	2.32 (2.20-2.45)
HTN	3.02 (2.99-3.06)	3.21 (3.18-3.25)	1.78 (1.69-1.87)	2.05 (1.97-2.13)
**Number of CMDs**				
0	1.00 (Ref)	1.00 (Ref)	1.00 (Ref)	1.00 (Ref)
1	1.21 (1.20-1.21)	1.15 (1.15-1.16)	1.23 (1.21-1.26)	1.30 (1.28-1.32)
2	3.69 (3.65-3.74)	3.37 (3.33-3.40)	2.19 (2.09-2.31)	2.45 (2.35-2.55)
3	7.82 (7.51-8.13)	6.75 (6.55-6.96)	3.20 (2.71-3.79)	3.91 (3.51-4.34)
**Patterns of CMDs**				
No CMDs	1.00 (Ref)	1.00 (Ref)	1.00 (Ref)	1.00 (Ref)
Only underweight	1.39 (1.39-1.39)	1.24 (1.23-1.25)	1.09 (1.04-1.14)	1.03 (0.98-1.08)
Only overweight	1.04 (1.04-1.04)	1.04 (1.03-1.04)	1.10 (1.08-1.13)	1.13 (1.11-1.16)
Only obese	1.29 (1.29-1.29)	1.22 (1.21-1.22)	1.37 (1.34-1.41)	1.48 (1.45-1.51)
Only DM	5.83 (5.82-5.83)	3.64 (3.50-3.77)	2.38 (2.04-2.78)	2.19 (1.87-2.56)
Only HTN	3.06 (3.06-3.06)	3.55 (3.45-3.65)	1.58 (1.38-1.80)	2.07 (1.85-2.31)
Underweight + DM	5.12 (5.10-5.14)	3.92 (3.36-4.58)	3.23 (1.89-5.52)	2.00 (1.03-3.87)
Overweight + DM	5.93 (5.93-5.94)	3.42 (3.32-3.53)	2.07 (1.74-2.46)	2.43 (2.14-2.76)
Obese + DM	5.34 (5.33-5.34)	3.58 (3.51-3.65)	2.94 (2.65-3.26)	2.71 (2.51-2.94)
Underweight + HTN	4.09 (4.07-4.10)	4.34 (3.88-4.85)	2.05 (1.28-3.27)	3.07 (2.11-4.46)
Overweight + HTN	3.07 (3.06-3.07)	3.32 (3.24-3.39)	1.97 (1.75-2.21)	2.19 (2.00-2.40)
Obese + HTN	3.36 (3.35-3.36)	3.20 (3.15-3.24)	2.04 (1.90-2.19)	2.41 (2.28-2.55)
DM + HTN	0.01 (0.01-0.01)	9.34 (8.40-10.39)	3.40 (2.12-5.46)	4.59 (3.26-6.47)
Underweight + DM + HTN	0.00 (0.00-1E122)	7.66 (4.65-12.59)	6.42 (0.86-47.73)	0.01 (0.00-29E13)
Overweight + DM + HTN	0.86 (0.85-0.86)	7.50 (6.98-8.07)	3.74 (2.56-5.46)	3.56 (2.75-4.62)
Obese + DM + HTN	4.87 (4.86-4.87)	6.59 (6.38-6.81)	3.08 (2.55-3.72)	4.03 (3.59-4.53)
**Gestational CMDs**				
**Individual CMDs**				
Non-ideal GWG				
Inadequate	1.60 (1.59-1.61)	1.49 (1.48-1.49)	2.01 (1.96-2.05)	1.87 (1.84-1.91)
Adequate	1.00 (Ref)	1.00 (Ref)	1.00 (Ref)	1.00 (Ref)
Excessive	0.80 (0.79-0.80)	0.83 (0.82-0.83)	0.82 (0.80-0.84)	0.82 (0.80-0.83)
GDM	1.69 (1.67-1.70)	1.56 (1.55-1.58)	0.90 (0.86-0.94)	0.82 (0.79-0.85)
HDP	3.07 (3.05-3.09)	3.24 (3.22-3.26)	1.13 (1.09-1.17)	1.14 (1.10-1.17)
**Number of CMDs**				

0	1.00 (Ref)	1.00 (Ref)	1.00 (Ref)	1.00 (Ref)
1	1.06 (1.05-1.06)	1.12 (1.11-1.12)	1.22 (1.20-1.25)	1.25 (1.23-1.28)
2	2.37 (2.35-2.39)	2.37 (2.35-2.38)	1.19 (1.14-1.23)	1.17 (1.13-1.21)
3	4.30 (4.22-4.39)	4.36 (4.28-4.44)	1.29 (1.14-1.45)	1.28 (1.15-1.41)
**Patterns of CMDs**				
No CMDs	1.00 (Ref)	1.00 (Ref)	1.00 (Ref)	1.00 (Ref)
Only inadequate GWG	1.68 (1.67-1.69)	1.57 (1.56-1.58)	2.07 (2.03-2.13)	1.94 (1.90-1.98)
Only excessive GWG	0.73 (0.72-0.73)	0.76 (0.76-0.77)	0.81 (0.79-0.83)	0.80 (0.78-0.82)
Only GDM	1.49 (1.46-1.52)	1.45 (1.43-1.47)	0.90 (0.82-0.99)	0.82 (0.76-0.89)
Only HDP	3.35 (3.30-3.39)	3.62 (3.57-3.67)	1.38 (1.29-1.48)	1.37 (1.29-1.46)
Inadequate GWG + GDM	1.82 (1.79-1.85)	1.74 (1.71-1.76)	1.30 (1.20-1.41)	1.17 (1.10-1.25)
Excessive GWG + GDM	1.41 (1.39-1.43)	1.38 (1.36-1.40)	0.87 (0.81-0.94)	0.81 (0.76-0.87)
Inadequate GWG + HDP	4.26 (4.19-4.33)	4.54 (4.48-4.61)	2.30 (2.15-2.47)	2.01 (1.90-2.13)
Excessive GWG + HDP	2.58 (2.56-2.61)	2.80 (2.77-2.83)	0.98 (0.93-1.04)	1.06 (1.00-1.11)
GDM + HDP	4.33 (4.19-4.48)	4.33 (4.20-4.46)	1.11 (0.89-1.39)	0.97 (0.80-1.18)
Inadequate GWG + GDM + HDP	4.43 (4.27-4.59)	4.66 (4.51-4.81)	1.65 (1.35-2.01)	1.61 (1.36-1.90)
Excessive GWG + GDM + HDP	4.13 (4.04-4.23)	4.12 (4.04-4.22)	1.11 (0.96-1.29)	1.10 (0.97-1.25)

Values were expressed as odds ratio (95% confidence interval).

* Separate models were built for each individual CMDs.

Models were adjusted for maternal age, marital status, educational level, parity, time of initiation of prenatal care, total number of prenatal care visits, smoking before or during pregnancy, infant sex.

CMD, cardiometabolic disorder; BMI, body mass index; DM, diabetes mellitus; HTN, hypertension; GWG, gestational weight gain; GDM, gestational diabetes mellitus; HDP, hypertensive disorders of pregnancy.

Table S11. Subgroup analysis for associations between pre-pregnancy cardiometabolic disorders and gestational cardiometabolic disorders with preterm birth and infant mortality by marital status.

	Preterm birth		Infant mortality	
Marital status	Married	Unmarried	Married	Unmarried
**Pre-pregnancy CMDs**				
**Individual CMDs***				
BMI				
Underweight	1.25 (1.24-1.27)	1.33 (1.32-1.35)	1.05 (0.99-1.11)	1.07 (1.03-1.12)
Normal weight	1.00 (Ref)	1.00 (Ref)	1.00 (Ref)	1.00 (Ref)
Overweight	1.14 (1.14-1.15)	0.95 (0.95-0.96)	1.16 (1.13-1.19)	1.07 (1.05-1.10)
Obese	1.54 (1.53-1.55)	1.09 (1.08-1.09)	1.50 (1.47-1.54)	1.34 (1.32-1.37)
DM	4.15 (4.09-4.21)	4.17 (4.10-4.23)	2.35 (2.21-2.51)	2.23 (2.11-2.36)
HTN	3.21 (3.17-3.24)	2.79 (2.76-2.82)	1.86 (1.77-1.95)	1.72 (1.65-1.79)
**Number of CMDs**				
0	1.00 (Ref)	1.00 (Ref)	1.00 (Ref)	1.00 (Ref)
1	1.29 (1.28-1.29)	1.03 (1.03-1.04)	1.29 (1.26-1.31)	1.20 (1.18-1.22)
2	3.86 (3.82-3.90)	2.90 (2.87-2.93)	2.32 (2.21-2.43)	2.03 (1.94-2.12)
3	7.57 (7.32-7.84)	6.12 (5.91-6.34)	3.50 (3.04-4.03)	3.11 (2.76-3.51)
**Patterns of CMDs**				
No CMDs	1.00 (Ref)	1.00 (Ref)	1.00 (Ref)	1.00 (Ref)
Only underweight	1.26 (1.25-1.28)	1.34 (1.34-1.34)	1.05 (0.99-1.11)	1.07 (1.02-1.12)
Only overweight	1.12 (1.11-1.12)	0.94 (0.94-0.94)	1.15 (1.12-1.18)	1.06 (1.04-1.09)
Only obese	1.45 (1.44-1.45)	1.02 (1.02-1.02)	1.46 (1.43-1.49)	1.31 (1.29-1.34)
Only DM	4.26 (4.11-4.41)	5.14 (5.14-5.15)	2.18 (1.84-2.58)	2.36 (2.04-2.74)
Only HTN	3.35 (3.26-3.44)	2.99 (2.98-2.99)	1.81 (1.59-2.07)	1.61 (1.43-1.80)
Underweight + DM	4.46 (3.78-5.25)	5.19 (5.17-5.20)	1.61 (0.66-3.89)	2.86 (1.73-4.72)
Overweight + DM	4.57 (4.43-4.72)	4.20 (4.20-4.20)	2.36 (2.03-2.74)	2.13 (1.84-2.47)
Obese + DM	4.61 (4.51-4.71)	3.59 (3.59-3.59)	2.99 (2.73-3.28)	2.46 (2.25-2.69)
Underweight + HTN	4.33 (3.82-4.91)	3.73 (3.73-3.74)	1.64 (0.87-3.07)	2.62 (1.87-3.66)
Overweight + HTN	3.35 (3.28-3.43)	2.77 (2.77-2.77)	2.04 (1.83-2.28)	1.82 (1.65-2.00)
Obese + HTN	3.63 (3.58-3.69)	2.56 (2.56-2.56)	2.19 (2.04-2.34)	1.90 (1.79-2.01)
DM + HTN	9.64 (8.57-10.85)	1.44 (1.44-1.45)	3.64 (2.24-5.93)	4.10 (2.94-5.73)
Underweight + DM + HTN	6.76 (3.61-12.67)	579E9 (0.00-I)	4.29 (0.56-33.20)	0.01 (0.00-42E14)
Overweight + DM + HTN	8.51 (7.84-9.23)	3.10 (3.10-3.11)	3.36 (2.38-4.74)	3.30 (2.49-4.37)
Obese + DM + HTN	7.43 (7.16-7.72)	4.81 (4.81-4.82)	3.56 (3.05-4.15)	3.10 (2.72-3.54)
**Gestational CMDs**				
**Individual CMDs**				
GWG				
Inadequate	1.53 (1.53-1.54)	1.51 (1.50-1.52)	2.04 (2.00-2.09)	1.79 (1.76-1.83)
Adequate	1.00 (Ref)	1.00 (Ref)	1.00 (Ref)	1.00 (Ref)
Excessive	0.84 (0.83-0.84)	0.76 (0.76-0.77)	0.80 (0.78-0.82)	0.79 (0.78-0.81)
GDM	1.71 (1.69-1.72)	1.56 (1.54-1.57)	0.87 (0.83-0.90)	0.87 (0.83-0.90)
HDP	3.36 (3.33-3.38)	2.80 (2.79-2.82)	1.20 (1.16-1.24)	1.02 (0.99-1.05) [c]
**Number of CMDs**				

0	1.00 (Ref)	1.00 (Ref)	1.00 (Ref)	1.00 (Ref)
1	1.10 (1.09-1.10)	1.05 (1.04-1.05)	1.24 (1.22-1.27)	1.19 (1.17-1.22)
2	2.49 (2.47-2.50)	2.18 (2.17-2.20)	1.24 (1.19-1.28)	1.07 (1.03-1.11)
3	4.75 (4.67-4.84)	3.70 (3.62-3.78)	1.28 (1.14-1.43)	1.21 (1.08-1.35)
**Patterns of CMDs**				
No CMDs	1.00 (Ref)	1.00 (Ref)	1.00 (Ref)	1.00 (Ref)
Only inadequate GWG	1.61 (1.60-1.62)	1.59 (1.58-1.60)	2.11 (2.06-2.16)	1.85 (1.81-1.89)
Only excessive GWG	0.77 (0.76-0.77)	0.70 (0.69-0.70)	0.78 (0.76-0.80)	0.79 (0.77-0.81)
Only GDM	1.55 (1.53-1.58)	1.41 (1.39-1.44)	0.84 (0.77-0.91)	0.91 (0.83-0.99)
Only HDP	3.70 (3.65-3.75)	3.03 (2.99-3.08)	1.43 (1.34-1.54)	1.23 (1.15-1.31)
Inadequate GWG + GDM	1.90 (1.88-1.93)	1.67 (1.64-1.70)	1.35 (1.27-1.45)	1.19 (1.10-1.29)
Excessive GWG + GDM	1.49 (1.47-1.51)	1.27 (1.25-1.29)	0.82 (0.76-0.89)	0.80 (0.75-0.86)
Inadequate GWG + HDP	4.76 (4.69-4.84)	3.73 (3.67-3.79)	2.48 (2.32-2.65)	1.69 (1.58-1.79)
Excessive GWG + HDP	2.88 (2.86-2.91)	2.33 (2.31-2.36)	1.05 (0.99-1.11)	0.91 (0.87-0.96)
GDM + HDP	4.63 (4.50-4.77)	3.80 (3.66-3.95)	1.09 (0.89-1.33)	0.93 (0.74-1.16)
Inadequate GWG + GDM + HDP	4.94 (4.79-5.10)	3.94 (3.78-4.11)	1.60 (1.34-1.91)	1.59 (1.31-1.91)
Excessive GWG + GDM + HDP	4.60 (4.50-4.70)	3.47 (3.38-3.55)	1.11 (0.96-1.28)	1.03 (0.90-1.18)

Values were expressed as odds ratio (95% confidence interval).

* Separate models were built for each individual CMDs.

Models were adjusted for maternal age, race/ethnicity, educational level, parity, time of initiation of prenatal care, total number of prenatal care visits, smoking before or during pregnancy, infant sex.

CMD, cardiometabolic disorder; BMI, body mass index; DM, diabetes mellitus; HTN, hypertension; GWG, gestational weight gain; GDM, gestational diabetes mellitus; HDP, hypertensive disorders of pregnancy.

Table S12. Subgroup analysis for associations between pre-pregnancy cardiometabolic disorders and gestational cardiometabolic disorders with preterm birth and infant mortality by educational level.

	Preterm birth		Infant mortality	
Educational level	Under college	College or above	Under college	College or above
**Pre-pregnancy CMDs**				
**Individual CMDs***				
BMI				
Underweight	1.35 (1.33-1.36)	1.16 (1.14-1.17)	1.08 (1.04-1.12)	1.00 (0.92-1.08)
Normal weight	1.00 (Ref)	1.00 (Ref)	1.00 (Ref)	1.00 (Ref)
Overweight	0.99 (0.98-0.99)	1.18 (1.17-1.18)	1.09 (1.07-1.11)	1.18 (1.15-1.22)
Obese	1.18 (1.18-1.19)	1.61 (1.60-1.62)	1.35 (1.32-1.37)	1.62 (1.58-1.67)
DM	4.17 (4.12-4.22)	3.98 (3.90-4.06)	2.34 (2.23-2.45)	2.13 (1.94-2.34)
HTN	2.94 (2.91-2.97)	3.14 (3.10-3.18)	1.72 (1.66-1.78)	1.97 (1.85-2.09)
**Number of CMDs**				
0	1.00 (Ref)	1.00 (Ref)	1.00 (Ref)	1.00 (Ref)
1	1.09 (1.08-1.09)	1.32 (1.31-1.32)	1.21 (1.19-1.22)	1.33 (1.29-1.36)
2	3.18 (3.16-3.21)	3.77 (3.72-3.82)	2.05 (1.98-2.13)	2.49 (2.35-2.65)
3	6.53 (6.35-6.72)	7.53 (7.19-7.88)	3.37 (3.04-3.72)	2.98 (2.41-3.70)
**Patterns of CMDs**				
No CMDs	1.00 (Ref)	1.00 (Ref)	1.00 (Ref)	1.00 (Ref)
Only underweight	1.35 (1.35-1.35)	1.17 (1.15-1.18)	1.08 (1.04-1.12)	1.00 (0.92-1.08)
Only overweight	0.97 (0.97-0.97)	1.15 (1.14-1.16)	1.08 (1.06-1.10)	1.16 (1.13-1.20)
Only obese	1.11 (1.11-1.11)	1.52 (1.51-1.53)	1.32 (1.29-1.34)	1.57 (1.53-1.62)
Only DM	5.08 (5.08-5.09)	3.83 (3.67-4.00)	2.48 (2.20-2.81)	1.60 (1.23-2.07)
Only HTN	3.17 (3.16-3.17)	3.27 (3.17-3.38)	1.70 (1.54-1.88)	1.76 (1.50-2.07)
Underweight + DM	5.20 (5.19-5.22)	3.67 (2.95-4.57)	2.87 (1.85-4.43)	1.31 (0.32-5.27)
Overweight + DM	4.30 (4.30-4.30)	4.39 (4.22-4.57)	2.32 (2.06-2.60)	2.15 (1.73-2.67)
Obese + DM	3.88 (3.88-3.88)	4.53 (4.40-4.68)	2.58 (2.40-2.77)	3.08 (2.69-3.53)
Underweight + HTN	3.95 (3.94-3.96)	4.08 (3.47-4.79)	2.33 (1.68-3.22)	2.59 (1.28-5.25)
Overweight + HTN	2.95 (2.95-2.95)	3.39 (3.30-3.48)	1.76 (1.61-1.92)	2.40 (2.12-2.72)
Obese + HTN	2.86 (2.85-2.86)	3.62 (3.56-3.69)	1.88 (1.79-1.98)	2.45 (2.26-2.67)
DM + HTN	8.58 (8.56-8.60)	10.50 (9.07-12.15)	3.73 (2.73-5.09)	5.10 (2.79-9.32)
Underweight + DM + HTN	48E15 (0.00-I)	2.81 (0.92-8.57)	1.82 (0.25-13.28)	0.02 (0.00-88E22)
Overweight + DM + HTN	4.45 (4.44-4.46)	8.99 (8.08-10.01)	3.21 (2.51-4.11)	3.56 (2.22-5.73)
Obese + DM + HTN	5.30 (5.30-5.30)	7.33 (6.96-7.71)	3.41 (3.06-3.80)	2.92 (2.30-3.71)
**Gestational CMDs**				
**Individual CMDs**				
GWG				
Inadequate	1.49 (1.48-1.49)	1.59 (1.58-1.61)	1.80 (1.76-1.83)	2.22 (2.16-2.29)
Adequate	1.00 (Ref)	1.00 (Ref)	1.00 (Ref)	1.00 (Ref)
Excessive	0.79 (0.79-0.79)	0.83 (0.82-0.83)	0.81 (0.80-0.83)	0.77 (0.75-0.79)
GDM	1.61 (1.60-1.62)	1.70 (1.68-1.71)	0.88 (0.85-0.91)	0.86 (0.81-0.90)
HDP	2.99 (2.97-3.00)	3.34 (3.31-3.36)	1.06 (1.03-1.09)	1.20 (1.15-1.25)

**Number of CMDs**				
0	1.00 (Ref)	1.00 (Ref)	1.00 (Ref)	1.00 (Ref)
1	1.07 (1.07-1.08)	1.09 (1.08-1.10)	1.20 (1.18-1.22)	1.26 (1.22-1.29)
2	2.27 (2.25-2.28)	2.50 (2.48-2.53)	1.12 (1.09-1.15)	1.23 (1.17-1.29)
3	4.08 (4.01-4.15)	4.69 (4.58-4.79)	1.25 (1.14-1.37)	1.23 (1.05-1.44)
**Patterns of CMDs**				
No CMDs	1.00 (Ref)	1.00 (Ref)	1.00 (Ref)	1.00 (Ref)
Only inadequate GWG	1.56 (1.56-1.57)	1.67 (1.66-1.69)	1.84 (1.81-1.88)	2.34 (2.27-2.42)
Only excessive GWG	0.73 (0.72-0.73)	0.76 (0.75-0.76)	0.80 (0.78-0.82)	0.76 (0.73-0.78)
Only GDM	1.47 (1.45-1.49)	1.53 (1.51-1.56)	0.87 (0.81-0.94)	0.89 (0.80-0.99)
Only HDP	3.26 (3.22-3.30)	3.71 (3.66-3.77)	1.25 (1.18-1.32)	1.54 (1.41-1.68)
Inadequate GWG + GDM	1.73 (1.71-1.76)	1.93 (1.89-1.96)	1.23 (1.15-1.30)	1.33 (1.22-1.46)
Excessive GWG + GDM	1.35 (1.33-1.37)	1.47 (1.44-1.49)	0.83 (0.79-0.88)	0.81 (0.73-0.90)
Inadequate GWG + HDP	4.01 (3.96-4.06)	4.94 (4.85-5.03)	1.84 (1.74-1.94)	2.63 (2.41-2.86)
Excessive GWG + HDP	2.51 (2.49-2.53)	2.84 (2.81-2.87)	0.95 (0.91-0.99)	1.04 (0.97-1.12)
GDM + HDP	4.12 (4.00-4.24)	4.69 (4.52-4.86)	0.96 (0.80-1.15)	1.18 (0.91-1.53)
Inadequate GWG + GDM + HDP	4.28 (4.15-4.41)	4.96 (4.77-5.16)	1.57 (1.35-1.83)	1.65 (1.29-2.10)
Excessive GWG + GDM + HDP	3.86 (3.79-3.94)	4.51 (4.39-4.63)	1.08 (0.97-1.21)	1.04 (0.85-1.27)

Values were expressed as odds ratio (95% confidence interval).

* Separate models were built for each individual CMDs.

Models were adjusted for maternal age, race/ethnicity, marital status, parity, time of initiation of prenatal care, total number of prenatal care visits, smoking before or during pregnancy, infant sex.

CMD, cardiometabolic disorder; BMI, body mass index; DM, diabetes mellitus; HTN, hypertension; GWG, gestational weight gain; GDM, gestational diabetes mellitus; HDP, hypertensive disorders of pregnancy.

Table S13. Associations between individual and number of pre-pregnancy and gestational cardiometabolic disorders with preterm birth and infant mortality.

	**Preterm birth**	**Infant mortality**
**CMDs**	OR (95% CI)	OR (95% CI)
**Individual CMDs**[a]		
BMI		
Underweight	1.26 (1.25-1.27)	0.99 (0.96-1.03)
Normal weight	1.00 (Ref)	1.00 (Ref)
Overweight	1.13 (1.13-1.14)	1.33 (1.31-1.35)
Obese	1.16 (1.15-1.16)	1.56 (1.54-1.58)
DM	3.21 (3.17-3.24)	1.86 (1.79-1.95)
HTN	3.04 (3.02-3.07)	1.57 (1.52-1.62)
GWG		
Inadequate	1.53 (1.53-1.54)	1.92 (1.89-1.95)
Adequate	1.00 (Ref)	1.00 (Ref)
Excessive	0.74 (0.73-0.74)	0.74 (0.73-0.76)
GDM	1.40 (1.40-1.41)	0.77 (0.75-0.79)
HDP	3.30 (3.28-3.31)	1.12 (1.09-1.15)
**Number of CMDs**[b]		
0	1.00 (Ref)	1.00 (Ref)
1	1.26 (1.26-1.27)	1.56 (1.53-1.60)
2	1.33 (1.33-1.34)	1.71 (1.67-1.75)
3	2.96 (2.94-2.98)	1.86 (1.81-1.92)
4	6.09 (6.02-6.16)	2.36 (2.23-2.49)
Continuous[#]	1.39 (1.39-1.39)	1.19 (1.18-1.20)
P for trend	<0.0001	<0.0001

[a] Models includes all six CMDs in one model, adjusted for maternal age, race/ethnicity, marital status, educational level, parity, time of initiation of prenatal care, total number of prenatal care visits, smoking before or during pregnancy, infant sex.

[b] Models were adjusted for maternal age, race/ethnicity, marital status, educational level, parity, time of initiation of prenatal care, total number of prenatal care visits, smoking before or during pregnancy, infant sex.

[#] Models were treated using total CMD number as a continuous variable.

CMD, cardiometabolic disorder; BMI, body mass index; DM, diabetes mellitus; HTN, hypertension; GWG, gestational weight gain; GDM, gestational diabetes mellitus; HDP, hypertensive disorders of pregnancy.

Table S14. Comparison of the preterm birth and infant mortality between combinations of pre-pregnancy and gestational CMDs patterns.

BMI	GWG	Diabetes	Hypertension	n (%)	Preterm birth (Yes), n (%)	Infant mortality (Yes), n (%)
normal	adequate	No	No	3,723,376 (15.2)	205,976 (5.5)	9,442 (0.3)
normal	adequate	No	HTN	22,284 (0.1)	4,091 (18.4)	130 (0.6)
normal	adequate	No	HDP	122,059 (0.5)	22,632 (18.5)	524 (0.4)
normal	adequate	DM	No	11,077 (0.1)	2,006 (18.1)	67 (0.6)
normal	adequate	DM	HTN	824 (0.0)	355 (43.1)	17 (2.1)
normal	adequate	DM	HDP	1,535 (0.0)	734 (47.8)	13 (0.9)
normal	adequate	GDM	No	133,823 (0.6)	9,574 (7.2)	274 (0.2)
normal	adequate	GDM	HTN	1,893 (0.0)	390 (20.6)	12 (0.6)
normal	adequate	GDM	HDP	8,538 (0.0)	1,817 (21.3)	31 (0.4)
normal	inadequate	No	No	2,467,376 (10.1)	277,255 (11.2)	18,838 (0.8)
normal	inadequate	No	HTN	16,689 (0.1)	5,134 (30.8)	274 (1.6)
normal	inadequate	No	HDP	72,497 (0.3)	21,100 (29.1)	804 (1.1)
normal	inadequate	DM	No	10,428 (0.0)	2,583 (24.8)	149 (1.4)
normal	inadequate	DM	HTN	890 (0.0)	**470 (52.8)**	20 (2.3)
normal	inadequate	DM	HDP	1,213 (0.0)	**688 (56.7)**	22 (1.8)
normal	inadequate	GDM	No	138,748 (0.6)	14,173 (10.2)	436 (0.3)
normal	inadequate	GDM	HTN	1,968 (0.0)	550 (28.0)	15 (0.8)
normal	inadequate	GDM	HDP	7,396 (0.0)	1,933 (26.1)	42 (0.6)
normal	excessive	No	No	3,478,257 (14.2)	129,340 (3.7)	7,373 (0.2)
normal	excessive	No	HTN	26,397 (0.1)	3,962 (15.0)	126 (0.5)
normal	excessive	No	HDP	190,444 (0.8)	26,376 (13.9)	623 (0.3)
normal	excessive	DM	No	11,564 (0.1)	1,975 (17.1)	62 (0.5)
normal	excessive	DM	HTN	991 (0.0)	384 (38.8)	17 (1.7)
normal	excessive	DM	HDP	2,418 (0.0)	1,141 (47.2)	18 (0.7)
normal	excessive	GDM	No	95,845 (0.4)	6,237 (6.5)	218 (0.2)
normal	excessive	GDM	HTN	1,875 (0.0)	353 (18.8)	08 (0.4)
normal	excessive	GDM	HDP	10,218 (0.0)	2,019 (19.8)	37 (0.4)
underweight	adequate	No	No	323,733 (1.3)	21,515 (6.7)	894 (0.3)
underweight	adequate	No	HTN	1,166 (0.0)	273 (23.4)	9 (0.8)
underweight	adequate	No	HDP	8,389 (0.0)	1,746 (20.8)	37 (0.4)
underweight	adequate	DM	No	591 (0.0)	124 (21.0)	1 (0.2)
underweight	adequate	DM	HTN	28 (0.0)	10 (35.7)	0 (0.0)
underweight	adequate	DM	HDP	66 (0.0)	32 (48.5)	0 (0.0)
underweight	adequate	GDM	No	8,696 (0.0)	638 (7.3)	17 (0.2)
underweight	adequate	GDM	HTN	50 (0.0)	7 (14.0)	0 (0.0)
underweight	adequate	GDM	HDP	417 (0.0)	80 (19.2)	1 (0.2)
underweight	inadequate	No	No	262,037 (1.1)	38,774 (14.8)	2,233 (0.9)
underweight	inadequate	No	HTN	1,139 (0.0)	418 (36.7)	29 (2.6)
underweight	inadequate	No	HDP	6,126 (0.0)	2,087 (34.1)	64 (1.0)
underweight	inadequate	DM	No	666 (0.0)	195 (29.3)	14 (2.1)

underweight	inadequate	DM	HTN	42 (0.0)	20 (47.6)	1 (2.4)
underweight	inadequate	DM	HDP	62 (0.0)	**43 (69.4)**	**4 (6.5)**
underweight	inadequate	GDM	No	10,191 (0.0)	1,280 (12.6)	32 (0.3)
underweight	inadequate	GDM	HTN	88 (0.0)	24 (27.3)	0 (0.0)
underweight	inadequate	GDM	HDP	398 (0.0)	113 (28.4)	5 (1.3)
underweight	excessive	No	No	169,872 (0.7)	8,096 (4.8)	537 (0.3)
underweight	excessive	No	HTN	924 (0.0)	166 (18.0)	9 (1.0)
underweight	excessive	No	HDP	7,610 (0.0)	1,225 (16.1)	32 (0.4)
underweight	excessive	DM	No	448 (0.0)	81 (18.1)	2 (0.5)
underweight	excessive	DM	HTN	38 (0.0)	18 (47.4)	0 (0.0)
underweight	excessive	DM	HDP	85 (0.0)	38 (44.7)	2 (2.4)
underweight	excessive	GDM	No	4,050 (0.0)	325 (8.0)	9 (0.2)
underweight	excessive	GDM	HTN	69 (0.0)	13 (18.8)	0 (0.0)
underweight	excessive	GDM	HDP	351 (0.0)	68 (19.4)	3 (0.9)
overweight	adequate	No	No	1,493,684 (6.1)	113,578 (7.6)	6,614 (0.4)
overweight	adequate	No	HTN	20,699 (0.1)	4,591 (22.2)	199 (1.0)
overweight	adequate	No	HDP	75,110 (0.3)	16,769 (22.3)	476 (0.6)
overweight	adequate	DM	No	10,806 (0.0)	2,042 (18.9)	83 (0.8)
overweight	adequate	DM	HTN	1,480 (0.0)	605 (40.9)	26 (1.8)
overweight	adequate	DM	HDP	1,834 (0.0)	802 (43.7)	25 (1.4)
overweight	adequate	GDM	No	118,071 (0.5)	10,431 (8.8)	284 (0.2)
overweight	adequate	GDM	HTN	3,626 (0.0)	807 (22.3)	18 (0.5)
overweight	adequate	GDM	HDP	11,283 (0.1)	2,676 (23.7)	44 (0.4)
overweight	inadequate	No	No	756,000 (3.1)	85,913 (11.4)	8,087 (1.1)
overweight	inadequate	No	HTN	10,854 (0.0)	3,372 (31.1)	265 (2.4)
overweight	inadequate	No	HDP	32,057 (0.1)	9,039 (28.2)	417 (1.3)
overweight	inadequate	DM	No	6,940 (0.0)	1,580 (22.8)	102 (1.5)
overweight	inadequate	DM	HTN	991 (0.0)	444 (44.8)	20 (2.0)
overweight	inadequate	DM	HDP	995 (0.0)	483 (48.5)	17 (1.7)
overweight	inadequate	GDM	No	73,315 (0.3)	8,007 (10.9)	294 (0.4)
overweight	inadequate	GDM	HTN	2,411 (0.0)	616 (25.6)	18 (0.8)
overweight	inadequate	GDM	HDP	5,951 (0.0)	1,564 (26.3)	30 (0.5)
overweight	excessive	No	No	3,300,455 (13.5)	154,645 (4.7)	8,756 (0.3)
overweight	excessive	No	HTN	50,557 (0.2)	8,006 (15.8)	281 (0.6)
overweight	excessive	No	HDP	257,269 (1.1)	38,681 (15.0)	881 (0.3)
overweight	excessive	DM	No	20,232 (0.1)	3,543 (17.5)	118 (0.6)
overweight	excessive	DM	HTN	2,865 (0.0)	1,070 (37.4)	43 (1.5)
overweight	excessive	DM	HDP	4,908 (0.0)	2,150 (43.8)	40 (0.8)
overweight	excessive	GDM	No	170,277 (0.7)	12,793 (7.5)	399 (0.2)
overweight	excessive	GDM	HTN	6,154 (0.0)	1,290 (21.0)	26 (0.4)
overweight	excessive	GDM	HDP	24,607 (0.1)	5,216 (21.2)	87 (0.4)
obese	adequate	No	No	1,213,082 (5.0)	95,560 (7.9)	7,156 (0.6)
obese	adequate	No	HTN	49,560 (0.2)	9,808 (19.8)	503 (1.0)
obese	adequate	No	HDP	120,688 (0.5)	24,059 (19.9)	781 (0.7)

obese	adequate	DM	No	19,039 (0.1)	3,448 (18.1)	191 (1.0)
obese	adequate	DM	HTN	6,468 (0.0)	2,196 (34.0)	116 (1.8)
obese	adequate	DM	HDP	4,683 (0.0)	1,821 (38.9)	43 (0.9)
obese	adequate	GDM	No	148,463 (0.6)	14,377 (9.7)	498 (0.3)
obese	adequate	GDM	HTN	13,425 (0.1)	3,003 (22.4)	89 (0.7)
obese	adequate	GDM	HDP	28,719 (0.1)	6,790 (23.6)	107 (0.4)
obese	inadequate	No	No	1,062,383 (4.4)	102,573 (9.7)	10,954 (1.0)
obese	inadequate	No	HTN	47,865 (0.2)	10,450 (21.8)	703 (1.5)
obese	inadequate	No	HDP	94,783 (0.4)	20,430 (21.6)	850 (0.9)
obese	inadequate	DM	No	19,535 (0.1)	3,813 (19.5)	292 (1.5)
obese	inadequate	DM	HTN	6,807 (0.0)	2,366 (34.8)	145 (2.1)
obese	inadequate	DM	HDP	4,050 (0.0)	1,569 (38.7)	54 (1.3)
obese	inadequate	GDM	No	155,707 (0.6)	16,067 (10.3)	745 (0.5)
obese	inadequate	GDM	HTN	15,404 (0.1)	3,222 (20.9)	114 (0.7)
obese	inadequate	GDM	HDP	26,896 (0.1)	6,248 (23.2)	170 (0.6)
obese	excessive	No	No	2,702,488 (11.1)	154,960 (5.7)	10,027 (0.4)
obese	excessive	No	HTN	112,318 (0.5)	19,078 (17.0)	755 (0.7)
obese	excessive	No	HDP	359,839 (1.5)	59,183 (16.5)	1,571 (0.4)
obese	excessive	DM	No	38,507 (0.2)	6,680 (17.4)	347 (0.9)
obese	excessive	DM	HTN	14,485 (0.1)	5,015 (34.6)	161 (1.1)
obese	excessive	DM	HDP	13,361 (0.1)	5,357 (40.1)	123 (0.9)
obese	excessive	GDM	No	248,437 (1.0)	22,241 (9.0)	790 (0.3)
obese	excessive	GDM	HTN	24,473 (0.1)	5,696 (23.3)	134 (0.6)
obese	excessive	GDM	HDP	65,018 (0.3)	15,336 (23.6)	295 (0.5)

BMI, body mass index; DM, diabetes mellitus; HTN, hypertension; GWG, gestational weight gain; GDM, gestational diabetes mellitus; HDP, hypertensive disorders of pregnancy.

Table S15. Associations of combinations of pre-pregnancy and gestational cardiometabolic disorders with preterm birth and infant mortality using Firth logistic regression.

BMI	GWG	Diabetes	Hyperte-nsion	All, n (%)		Preterm birth (Yes), n (%)			Infant mortality (Yes), n (%)		
				Total	Sampling	Total	Sampling	OR (95% CI)	Total	Sampling	OR (95% CI)
Ideal	Adequate	No	No	3,723,376 (15.23)	372,594 (15.24)	205,976 (5.53)	20,559 (5.52)	3.59 (3.21-4.01)	9,442 (0.25)	944 (0.25)	1.00 (ref)
Ideal	Adequate	No	HTN	22,284 (0.09)	2,260 (0.09)	4,091 (18.36)	422 (18.67)	3.80 (3.61-3.99)	130 (0.58)	12 (0.53)	1.69 (0.91-2.83)
Ideal	Adequate	No	HDP	122,059 (0.50)	12,181 (0.50)	22,632 (18.54)	2,310 (18.96)	4.01 (3.41-4.69)	524 (0.43)	52 (0.43)	1.44 (1.07-1.88)
Ideal	Adequate	DM	No	11,077 (0.05)	1,098 (0.04)	2,006 (18.11)	201 (18.31)	12.01 (8.64-16.64)	67 (0.60)	09 (0.82)	3.33 (1.62-5.98)
Ideal	Adequate	DM	HTN	824 (0.00)	85 (0.00)	355 (43.08)	40 (47.06)	15.01 (11.44-19.61)	17 (2.06)	1 (1.18)	4.19 (0.47-15.82)
Ideal	Adequate	DM	HDP	1,535 (0.01)	156 (0.01)	734 (47.82)	83 (53.21)	1.47 (1.38-1.58)	13 (0.85)	2 (1.28)	5.68 (1.18-16.39)
Ideal	Adequate	GDM	No	133,823 (0.55)	13,321 (0.54)	9,574 (7.15)	981 (7.36)	5.45 (3.85-7.58)	274 (0.20)	28 (0.21)	0.98 (0.66-1.39)
Ideal	Adequate	GDM	HTN	1,893 (0.01)	203 (0.01)	390 (20.60)	47 (23.15)	4.33 (3.64-5.12)	12 (0.63)	1 (0.49)	3.06 (0.35-11.04)
Ideal	Adequate	GDM	HDP	8,538 (0.03)	879 (0.04)	1,817 (21.28)	182 (20.71)	1.91 (1.88-1.95)	31 (0.36)	4 (0.46)	1.92 (0.64-4.31)
Ideal	Inadequate	No	No	2,467,376 (10.09)	246,412 (10.08)	277,255 (11.24)	27,568 (11.19)	5.75 (5.13-6.44)	18,838 (0.76)	1,895 (0.77)	2.38 (2.20-2.58)
Ideal	Inadequate	No	HTN	16,689 (0.07)	1,663 (0.07)	5,134 (30.76)	486 (29.22)	5.96 (5.64-6.30)	274 (1.64)	27 (1.62)	3.86 (2.55-5.57)
Ideal	Inadequate	No	HDP	72,497 (0.30)	7,309 (0.30)	21,100 (29.10)	2,124 (29.06)	5.16 (4.43-5.99)	804 (1.11)	77 (1.05)	2.70 (2.11-3.39)
Ideal	Inadequate	DM	No	10,428 (0.04)	1,028 (0.04)	2,583 (24.77)	247 (24.03)	a	149 (1.43)	15 (1.46)	4.84 (2.77-7.79)
Ideal	Inadequate	DM	HTN	890 (0.00)	88 (0.00)	470 (52.81)	45 (51.14)	b	20 (2.25)	2 (2.27)	6.97 (1.42-20.82)
Ideal	Inadequate	DM	HDP	1,213 (0.00)	130 (0.01)	688 (56.72)	75 (57.69)	2.05 (1.93-2.17)	22 (1.81)	5 (3.85)	10.37 (3.79-22.89)
Ideal	Inadequate	GDM	No	138,748 (0.57)	13,902 (0.57)	14,173 (10.21)	1,410 (10.14)	6.87 (4.93-9.45)	436 (0.31)	41 (0.29)	1.31 (0.95-1.77)
Ideal	Inadequate	GDM	HTN	1,968 (0.01)	196 (0.01)	550 (27.95)	56 (28.57)	5.41 (4.51-6.45)	15 (0.76)	3 (1.53)	6.44 (1.78-16.33)
Ideal	Inadequate	GDM	HDP	7,396 (0.03)	727 (0.03)	1,933 (26.14)	175 (24.07)	0.66 (0.65-0.68)	42 (0.57)	2 (0.28)	1.24 (0.26-3.51)
Ideal	Excessive	No	No	3,478,257 (14.23)	348,645 (14.26)	129,340 (3.72)	12,912 (3.70)	2.68 (2.39-2.99)	7,373 (0.21)	763 (0.22)	0.85 (0.77-0.94)
Ideal	Excessive	No	HTN	26,397 (0.11)	2,699 (0.11)	3,962 (15.01)	392 (14.52)	2.74 (2.62-2.86)	126 (0.48)	13 (0.48)	1.57 (0.87-2.58)
Ideal	Excessive	No	HDP	190,444 (0.78)	19,125 (0.78)	26,376 (13.85)	2,704 (14.14)	4.09 (3.49-4.77)	623 (0.33)	66 (0.35)	1.19 (0.92-1.51)
Ideal	Excessive	DM	No	11,564 (0.05)	1,163 (0.05)	1,975 (17.08)	206 (17.71)	12.89 (8.62-19.15)	62 (0.54)	3 (0.26)	1.19 (0.33-2.92)

Ideal	Excessive	DM	HTN	991 (0.00)	108 (0.00)	384 (38.75)	47 (43.52)	13.14 (10.06-17.12)	17 (1.72)	3 (2.78)	9.81 (2.69-25.13)
Ideal	Excessive	DM	HDP	2,418 (0.01)	246 (0.01)	1,141 (47.19)	106 (43.09)	1.26 (1.16-1.37)	18 (0.74)	2 (0.81)	3.14 (0.66-8.96)
Ideal	Excessive	GDM	No	95,845 (0.39)	9,573 (0.39)	6,237 (6.51)	637 (6.65)	3.62 (2.45-5.22)	218 (0.23)	25 (0.26)	1.08 (0.71-1.57)
Ideal	Excessive	GDM	HTN	1,875 (0.01)	189 (0.01)	353 (18.83)	36 (19.05)	4.31 (3.66-5.04)	8 (0.43)	0 (0.00)	0.79 (0.01-5.47)
Ideal	Excessive	GDM	HDP	10,218 (0.04)	1,006 (0.04)	2,019 (19.76)	207 (20.58)	1.32 (1.30-1.35)	37 (0.36)	5 (0.50)	1.86 (0.70-3.90)
Non-ideal	Adequate	No	No	3,030,499 (12.40)	303,586 (12.42)	230,653 (7.61)	23,239 (7.65)	4.16 (3.92-4.43)	14,664 (0.48)	1,549 (0.51)	1.78 (1.64-1.93)
Non-ideal	Adequate	No	HTN	71,425 (0.29)	7,090 (0.29)	14,672 (20.54)	1,502 (21.18)	4.15 (4.00-4.31)	711 (1.00)	56 (0.79)	2.41 (1.81-3.13)
Non-ideal	Adequate	No	HDP	204,187 (0.84)	20,325 (0.83)	42,574 (20.85)	4,195 (20.64)	4.16 (3.78-4.56)	1,294 (0.63)	133 (0.65)	2.10 (1.74-2.51)
Non-ideal	Adequate	DM	No	30,436 (0.12)	3,051 (0.12)	5,614 (18.45)	605 (19.83)	9.45 (8.09-11.02)	275 (0.90)	25 (0.82)	2.99 (1.96-4.36)
Non-ideal	Adequate	DM	HTN	7,976 (0.03)	772 (0.03)	2,811 (35.24)	284 (36.79)	12.23 (10.41-14.35)	142 (1.78)	14 (1.81)	5.71 (3.20-9.37)
Non-ideal	Adequate	DM	HDP	6,583 (0.03)	686 (0.03)	2,655 (40.33)	281 (40.96)	1.90 (1.81-1.98)	68 (1.03)	8 (1.17)	4.10 (1.90-7.63)
Non-ideal	Adequate	GDM	No	275,230 (1.13)	27,578 (1.13)	25,446 (9.25)	2,634 (9.55)	5.11 (4.53-5.76)	799 (0.29)	90 (0.33)	1.42 (1.14-1.75)
Non-ideal	Adequate	GDM	HTN	17,101 (0.07)	1,654 (0.07)	3,817 (22.32)	391 (23.64)	5.27 (4.88-5.70)	107 (0.63)	6 (0.36)	1.34 (0.55-2.68)
Non-ideal	Adequate	GDM	HDP	40,419 (0.17)	4,032 (0.16)	9,546 (23.62)	931 (23.09)	1.78 (1.75-1.82)	152 (0.38)	21 (0.52)	2.00 (1.26-2.99)
Non-ideal	Inadequate	No	No	2,080,420 (8.51)	207,420 (8.48)	227,260 (10.92)	22,413 (10.81)	4.67 (4.38-4.98)	21,274 (1.02)	2,160 (1.04)	3.07 (2.84-3.32)
Non-ideal	Inadequate	No	HTN	59,858 (0.24)	5,883 (0.24)	14,240 (23.79)	1,427 (24.26)	4.71 (4.51-4.93)	997 (1.67)	96 (1.63)	4.42 (3.54-5.44)
Non-ideal	Inadequate	No	HDP	132,966 (0.54)	13,132 (0.54)	31,556 (23.73)	3,154 (24.02)	4.52 (4.10-4.97)	1,331 (1.00)	126 (0.96)	2.63 (2.17-3.16)
Non-ideal	Inadequate	DM	No	27,141 (0.11)	2,742 (0.11)	5,588 (20.59)	598 (21.81)	8.75 (7.51-10.16)	408 (1.50)	44 (1.60)	5.44 (3.95-7.30)
Non-ideal	Inadequate	DM	HTN	7,840 (0.03)	815 (0.03)	2,830 (36.10)	300 (36.81)	11.88 (9.87-14.26)	166 (2.12)	18 (2.21)	6.20 (3.73-9.66)
Non-ideal	Inadequate	DM	HDP	5,107 (0.02)	523 (0.02)	2,095 (41.02)	218 (41.68)	2.10 (2.01-2.19)	75 (1.47)	9 (1.72)	5.48 (2.65-9.97)
Non-ideal	Inadequate	GDM	No	239,213 (0.98)	24,134 (0.99)	25,354 (10.60)	2,562 (10.62)	4.66 (4.12-5.24)	1,071 (0.45)	112 (0.46)	1.92 (1.57-2.33)
Non-ideal	Inadequate	GDM	HTN	17,903 (0.07)	1,750 (0.07)	3,862 (21.57)	371 (21.20)	5.65 (5.20-6.14)	132 (0.74)	11 (0.63)	2.47 (1.30-4.23)
Non-ideal	Inadequate	GDM	HDP	33,245 (0.14)	3,383 (0.14)	7,925 (23.84)	832 (24.59)	0.91 (0.89-0.92)	205 (0.62)	25 (0.74)	2.71 (1.77-3.94)
Non-ideal	Excessive	No	No	6,172,815 (25.25)	616,483 (25.22)	317,701 (5.15)	31,716 (5.14)	3.11 (2.98-3.26)	19,320 (0.31)	1,915 (0.31)	1.16 (1.08-1.26)
Non-ideal	Excessive	No	HTN	163,799 (0.67)	16,343 (0.67)	27,250 (16.64)	2,686 (16.44)	3.11 (3.03-3.20)	1,045 (0.64)	105 (0.64)	2.06 (1.67-2.51)
Non-ideal	Excessive	No	HDP	624,718 (2.56)	62,602 (2.56)	99,089 (15.86)	9,913 (15.83)	3.65 (3.39-3.92)	2,484 (0.40)	247 (0.39)	1.35 (1.17-1.55)

Non-ideal	Excessive	DM	No	59,187 (0.24)	5,888 (0.24)	10,304 (17.41)	1,006 (17.09)	8.60 (7.75-9.55)	467 (0.79)	34 (0.58)	2.22 (1.55-3.08)
Non-ideal	Excessive	DM	HTN	17,388 (0.07)	1,732 (0.07)	6,103 (35.10)	609 (35.16)	13.01 (11.78-14.36)	204 (1.17)	25 (1.44)	4.47 (2.91-6.53)
Non-ideal	Excessive	DM	HDP	18,354 (0.08)	1,815 (0.07)	7,545 (41.11)	753 (41.49)	1.59 (1.53-1.65)	165 (0.90)	16 (0.88)	3.13 (1.84-4.95)
Non-ideal	Excessive	GDM	No	422,764 (1.73)	42,282 (1.73)	35,359 (8.36)	3,522 (8.33)	5.08 (4.65-5.54)	1,198 (0.28)	114 (0.27)	1.10 (0.90-1.33)
Non-ideal	Excessive	GDM	HTN	30,696 (0.13)	3,132 (0.13)	6,999 (22.80)	719 (22.96)	5.05 (4.78-5.32)	160 (0.52)	16 (0.51)	1.89 (1.11-2.98)
Non-ideal	Excessive	GDM	HDP	89,976 (0.37)	8,968 (0.37)	20,620 (22.92)	2,023 (22.56)	3.59 (3.21-4.01)	385 (0.43)	33 (0.37)	1.37 (0.95-1.91)

a: For model stability and convergence, the patterns “DM, HTN, and inadequate GWG” and “DM, HTN, and excessive GWG” were combined into a single group.

b: For model stability and convergence, the patterns “DM, HDP, and inadequate GWG” and “DM, HDP, and excessive GWG” were combined into a single group.

BMI, body mass index; DM, diabetes mellitus; HTN, hypertension; GWG, gestational weight gain; GDM, gestational diabetes mellitus; HDP, hypertensive disorders of pregnancy.

S1 Checklist. STROBE Statement—Checklist of items that should be included in reports of ***cross-sectional studies.***

	Item No	Recommendation	Section and paragraph
**Title and abstract**	1	(*a*) Indicate the study's design with a commonly used term in the title or the abstract	Abstract
		(*b*) Provide in the abstract an informative and balanced summary of what was done and what was found	Abstract
**Introduction**			
Background/rationale	2	Explain the scientific background and rationale for the investigation being reported	Introduction, paragraph 1-4
Objectives	3	State specific objectives, including any prespecified hypotheses	Introduction, paragraph 5
**Methods**			
Study design	4	Present key elements of study design early in the paper	Methods, paragraph 1
Setting	5	Describe the setting, locations, and relevant dates, including periods of recruitment, exposure, follow-up, and data collection	Methods, paragraph 1-2
Participants	6	(*a*) Give the eligibility criteria, and the sources and methods of selection of participants	Methods, paragraph 2
Variables	7	Clearly define all outcomes, exposures, predictors, potential confounders, and effect modifiers. Give diagnostic criteria, if applicable	Methods, paragraph 3-8
Data sources/ measurement	8*	For each variable of interest, give sources of data and details of methods of assessment (measurement). Describe comparability of assessment methods if there is more than one group	Methods, paragraph 3-9
Bias	9	Describe any efforts to address potential sources of bias	NA
Study size	10	Explain how the study size was arrived at	Methods, paragraph 2
Quantitative variables	11	Explain how quantitative variables were handled in the analyses. If applicable, describe which groupings were chosen and why	Methods, paragraph 3-9
Statistical methods	12	(*a*) Describe all statistical methods, including those used to control for confounding	Statistical analysis, paragraph 1-3
		(*b*) Describe any methods used to examine subgroups and interactions	Statistical analysis, paragraph 3
		(*c*) Explain how missing data were addressed	Methods, paragraph 8
		(*d*) If applicable, describe analytical methods taking account of sampling strategy	NA
		(*e*) Describe any sensitivity analyses	Statistical analysis, paragraph 3

**Results**			
Participants	13*	(a) Report numbers of individuals at each stage of study—eg numbers potentially eligible, examined for eligibility, confirmed eligible, included in the study, completing follow-up, and analysed	Methods, paragraph 2
		(b) Give reasons for non-participation at each stage	Methods, paragraph 2
		(c) Consider use of a flow diagram	Figure 1
Descriptive data	14*	(a) Give characteristics of study participants (eg demographic, clinical, social) and information on exposures and potential confounders	Results, paragraph 1
		(b) Indicate number of participants with missing data for each variable of interest	Table 1
Outcome data	15*	Report numbers of outcome events or summary measures	Results, paragraph 1
Main results	16	(*a*) Give unadjusted estimates and, if applicable, confounder-adjusted estimates and their precision (eg, 95% confidence interval). Make clear which confounders were adjusted for and why they were included	Results, paragraph 2-9
		(*b*) Report category boundaries when continuous variables were categorized	NA
		(*c*) If relevant, consider translating estimates of relative risk into absolute risk for a meaningful time period	NA
Other analyses	17	Report other analyses done—eg analyses of subgroups and interactions, and sensitivity analyses	Results, paragraph 5,7,9
**Discussion**			
Key results	18	Summarise key results with reference to study objectives	Discussion, paragraph 1
Limitations	19	Discuss limitations of the study, taking into account sources of potential bias or imprecision. Discuss both direction and magnitude of any potential bias	Discussion, Strength and limitations
Interpretation	20	Give a cautious overall interpretation of results considering objectives, limitations, multiplicity of analyses, results from similar studies, and other relevant evidence	Discussion, Interpretation and comparison
Generalisability	21	Discuss the generalisability (external validity) of the study results	NA
**Other information**			
Funding	22	Give the source of funding and the role of the funders for the present study and, if applicable, for the original study on which the present article is based	Funding

*Give information separately for exposed and unexposed groups.
